# The Morphology of the Rat Vibrissal Array: A Model for Quantifying Spatiotemporal Patterns of Whisker-Object Contact

**DOI:** 10.1371/journal.pcbi.1001120

**Published:** 2011-04-07

**Authors:** R. Blythe Towal, Brian W. Quist, Venkatesh Gopal, Joseph H. Solomon, Mitra J. Z. Hartmann

**Affiliations:** 1 Department of Biomedical Engineering, Northwestern University, Evanston, Illinois, United States of America; 2 Department of Physics, Elmhurst College, Elmhurst, Illinois, United States of America; 3 Department of Mechanical Engineering, Northwestern University, Evanston, Illinois, United States of America; University College London, United Kingdom

## Abstract

In all sensory modalities, the data acquired by the nervous system is shaped by the biomechanics, material properties, and the morphology of the peripheral sensory organs. The rat vibrissal (whisker) system is one of the premier models in neuroscience to study the relationship between physical embodiment of the sensor array and the neural circuits underlying perception. To date, however, the three-dimensional morphology of the vibrissal array has not been characterized. Quantifying array morphology is important because it directly constrains the mechanosensory inputs that will be generated during behavior. These inputs in turn shape all subsequent neural processing in the vibrissal-trigeminal system, from the trigeminal ganglion to primary somatosensory (“barrel”) cortex. Here we develop a set of equations for the morphology of the vibrissal array that accurately describes the location of every point on every whisker to within ±5% of the whisker length. Given only a whisker's identity (row and column location within the array), the equations establish the whisker's two-dimensional (2D) shape as well as three-dimensional (3D) position and orientation. The equations were developed via parameterization of 2D and 3D scans of six rat vibrissal arrays, and the parameters were specifically chosen to be consistent with those commonly measured in behavioral studies. The final morphological model was used to simulate the contact patterns that would be generated as a rat uses its whiskers to tactually explore objects with varying curvatures. The simulations demonstrate that altering the morphology of the array changes the relationship between the sensory signals acquired and the curvature of the object. The morphology of the vibrissal array thus directly constrains the nature of the neural computations that can be associated with extraction of a particular object feature. These results illustrate the key role that the physical embodiment of the sensor array plays in the sensing process.

## Author Summary

Animals move in order to sense the world. Sensing is thus an active process, constrained by muscle biomechanics and by the material, shape, and structure of the sensing organs. The rat vibrissal system provides an ideal model to examine how the physical embodiment of a sensory array shapes the sensing process. Rats have approximately thirty macrovibrissae (whiskers) arranged in rows and columns on each side of their face. They brush their whiskers against objects to tactually extract object features. To date, however, the three-dimensional shape of the whisker array has not been characterized. We scanned six rats to develop equations for the complete structure of the whisker array. Given only a whisker's row and column identity, the equations establish the whisker's two-dimensional shape and three-dimensional position and orientation. We used this equation-based model to simulate the whisker-object contact patterns that would be generated as a rat uses its whiskers to tactually explore objects with varying curvatures. Altering the shape of the array dramatically altered the relationship between the simulated sensory input and object curvature. The structure of the whisker array thus directly constrains spatiotemporal input patterns and thereby, the nature of the neural processing associated with extraction of particular object features.

## Introduction

Animals use movements to acquire and refine incoming sensory data as they explore and navigate the environment. This means that – except under rare conditions most often found in the laboratory – sensing is an active process, constrained and shaped by the biomechanics of the muscles and by the material properties and morphology of the sensing organs. It is impossible to meaningfully characterize sensory input to the nervous system during active behavior without considering the physical embodiment of the sensor array.

The rat vibrissal (whisker) system is one of the oldest models in neuroscience for studying sensorimotor integration and active sensing [1], [2]. Approximately 30 macrovibrissae are arranged in a regular array on each side of the rat's face [3]. Rats move their whiskers at frequencies between 5–25 Hz to acquire tactile information about objects in the environment, including size, shape, orientation, and texture [4], [5], [6], [7], [8].

The morphology of the vibrissal array directly constrains the spatiotemporal patterns of mechanosensory inputs that will be generated as the rat actively explores an object. These patterns of whisker-object contact in turn shape all subsequent patterns of neural activation along the vibrissal-trigeminal pathway, from the brainstem to primary somatosensory (“barrel”) cortex. To date, however, the shape and structure of the rat vibrissal array has not been quantified, and there is thus no rigorous way to predict the input patterns that will occur during a given exploratory sequence.

The present study was undertaken to quantify the morphology of the rat vibrissal array and demonstrate its influence on the whisker-object contact patterns associated with tactile exploration of an object. A set of equations is developed that describes every point on every whisker in the entire vibrissal array. Given only a whisker's identity (that is, its row and column within the array), the equations establish the whisker's two dimensional (2D) shape as well as three-dimensional (3D) position and orientation. The final result is a model that accurately describes the 3D location of every point along a whisker to within 5% of the point's distance from the whisker base.

Simulations demonstrate that alterations in array morphology dramatically alter the mechanosensory signals that the rat would obtain as it whisks against an object. Specifically, for a particular head orientation, the average angle at which the whiskers contact a cylindrical object is uniquely related to the radius of the cylinder. The nervous system could potentially learn this relationship to allow the rat to determine object radius within the time span of a single whisk. If the morphology of the array is altered, however, the same head orientation no longer produces a unique relationship between average angle of contact and object radius.

The morphology of the vibrissal array thus directly modulates the information available to the nervous system and constrains the nature of the computations that can be associated with extraction of a particular object feature. These results underscore the critical importance of physical embodiment in the sensing process.

## Results

We describe a morphologically accurate 3D model of the vibrissal array. Parameters of the model were determined from 158 whiskers scanned in both 3D and 2D, and an additional 196 whiskers scanned in 2D.

Results are presented in five sections. First, a standard coordinate system for the head and whisker array is established. Second, relationships are identified between whisker parameters (shape, base-point location, and orientation) and whisker identity (i.e., the whisker's row and column within the array). Third, these relationships are quantified in a set of equations, subsequently used to generate the final model of the vibrissal array. Fourth, error analysis is performed to demonstrate that the positions of the whiskers in the model are accurate to within less than 5% of the whisker length. Finally, simulations using the model shed light on how the morphology of the array directly constrains the neural computations that could allow the rat to extract information about the surface curvature of an object.

### Standard position and orientation of the head

The heads and vibrissal arrays of three rats were scanned in a 3D volumetric scanner (see Materials and Methods).

**Figure 1:**
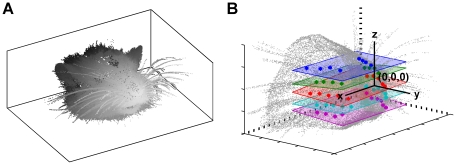
3D head reference frame. (**A**) Representative point cloud obtained from the 3D scanner. (**B**) Standard position and orientation for the head. The rat's snout is placed at the origin, and the rostrocaudal midline is collinear with the y-axis. Base points of whiskers in each row are aligned in planes (colored by row) that lie parallel to the *xy*-plane on average.

In post-processing, the 3D point cloud of the rat's head (Figure 1A) was placed in a standard position and orientation, defined using three criteria. First, the rat's nose (the centroid of the two nostrils) was defined to lie at the origin *(0,0,0)*. Second, the “rostrocaudal midline” of the head was required to lie in the *yz*-plane, with the caudal-to-rostral vector pointing in the positive *y*-direction. The rostrocaudal midline was defined as the line between the mean coordinate of all macrovibrissal base-points and the origin. Finally, the “whisker row planes” were forced to lie parallel to the *xy*-plane (Figure 1B).

For each row, the “whisker row plane” was defined as the best-fit plane to all whisker base-points within that row on both left and right sides of the face. The normal to each whisker row plane was averaged across all rows to obtain a single direction vector. Greek whiskers were omitted when computing best-fit planes. The maximum sum-squared-error across all planes was 1.59 mm^2^ (maximum residual was 0.86 mm). For each rat, the whisker row planes were all closely parallel (mean angle between normal vectors = 6.86°, maximum difference = 17.92°). Averaging the normal vectors of all best fit planes for a given rat yielded the head orientation that aligned the average row-plane normal vector with the positive *z*-axis. This ensured that all five planes were oriented as close as possible to parallel to the *xy*-plane.

### Relationships between whisker parameters and whisker identity

#### Parameterization of the vibrissal array

To pool data across rats, data were parameterized in terms of variables that are relatively easy to measure in behavioral studies. This parameterization relies on seven parameters specific to the mystacial pad and eight parameters specific to each whisker, listed in Table 1.

**Table 1:** Vibrissal array parameters.

	Category	Parameter	Variable
**Mystacial Pad** a	Position	Center (x,y,z)	***c***
	Shape	Major Radius	*r_a_*
		Semi-major Radius	*r_b_*
		Minor Radius	*r_c_*
	Orientation	Ellipsoid Theta	*θ_mp_*
		Ellipsoid Phi	*ϕ_mp_*
		Ellipsoid Psi	*ψ_mp_*
**Whisker** b	Position^c^	Base point Theta	*θ_BP_*
		Base point Phi	*ϕ_BP_*
	Shape	Arc length	*s*
		Coefficient of the quadratic term	*a*
	Orientation	Orientation Theta	*θ*
		Orientation Phi	*ϕ*
		Orientation Psi	*ψ*
		Orientation Zeta	*ζ*

Ellipsoid orientation angles of the mystacial pad (*θ_mp_ ϕ_mp_*, and *ψ_mp_*) are Euler angles. Base point position angles of the whiskers (*θ_BP_* and *ϕ_BP_*) are in spherical coordinates relative to the mystacial ellipsoid reference frame. Whisker orientation angles are projection angles, with the exception of *ζ*, which is the angle about the whisker's own axis. *mp* – mystacial pad, *BP* – base-point.

a modeled as an ellipsoid,

b modeled as a parabola,

c relative to mystacial ellipsoid.

#### The mystacial pad is modeled as an ellipsoid by fitting the whisker base-points

**Figure 2:**
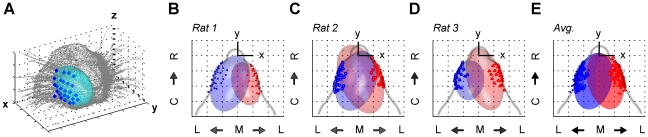
Ellipsoidal fits to the mystacial pad. (**A**) An example from one rat of the best-fit ellipsoid to whisker base-points on the right side of the mystacial pad. Whisker base-points are shown in blue and the best-fit ellipsoid in cyan. The rat head is placed in the standard position and orientation, and the view for this figure was chosen so as to best illustrate the mystacial pad. (**B–D**) Aerial view of the mystacial pad ellipsoid fits for each of the three rats scanned in 3D. Note the close fits to the contours of the rat's cheek (gray curve). (**E**) Model ellipsoid obtained from ellipsoids averaged across all rats. All base-points from all rats scanned in 3D are shown. For all plots B–E, left array base-points and best-fit ellipsoids are in blue; right array base-points and best-fit ellipsoids are in red; and grid lines represent 5 mm increments on all axes.

The shape of the mystacial pad was modeled as an ellipsoid (Figure 2A) whose three radii (major radius, semi-major radius, and minor radius) can be independently varied to effect changes in the mystacial curvature. This flexibility is important because the mystacial pad changes shape during the whisk cycle [9]. The results of ellipsoid fitting procedures are shown for all three rats in Figure 2B–2D. All six mystacial pad fits produced an r^2^ value of at least 0.53 (maximum of 0.88) and the maximum residual for any fit was 2.69 mm.

To obtain an average of all the ellipsoid parameters, ellipsoids from the left side were first reflected about the *y*-axis to eliminate sign differences between the two sides of the face. Parameters for all ellipsoids were then averaged together to produce the average model ellipsoids shown in Figure 2E. Final values for the averaged ellipsoid parameters are listed in Table 2.

**Table 2:** Mystacial pad ellipsoid parameter values.

Parameter name	Parameter variable	Averaged Parameter Value	Parameter Value Standard Error
Center (x,y,z)	***c***	[1.91, −7.65, −5.44] mm	±[0.41, 0.27, 0.60] mm
Major Radius	*r_a_*	9.53 mm	±0.79 mm
Semi-major Radius	*r_b_*	5.53 mm	±0.36 mm
Minor Radius	*r_c_*	6.97 mm	±0.41 mm
Ellipsoid Theta	*θ_mp_*	106.5°	±4.2°
Ellipsoid Phi	*ϕ_mp_*	−2.5°	±6.8°
Ellipsoid Psi	*ψ_mp_*	−19.5°	±4.9°

All values are relative to the standard head reference frame (Figure 1) and the fitting procedure is described in detail in *Materials and Methods*.

#### Positions of whisker base-points – dependence on row and column

**Figure 3:**
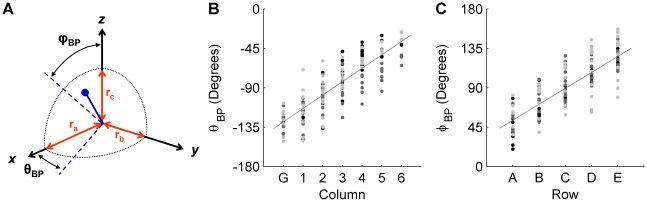
Base-point locations of whiskers vary with row and column. (**A**) Schematic illustrating the conversion of the (*x,y,z*) base-point location to mystacial pad ellipsoid coordinates (*r_BP_, ϕ_BP_*, and *θ_BP_*). Each base-point was constrained to lie on the ellipsoid surface (blue circle). The axis-aligned ellipsoid radii *(r_a_, r_b_, r_c_)* are shown in red. (**B**) The base-point *θ_BP_* was strongly correlated with whisker column. (**C**) The base-point *ϕ_BP_* was strongly correlated with whisker row. For both B and C, different marker colors (black, gray, and light gray) indicate rat of origin and indicate that no significant differences were found across rats.

The (*x,y,z*) positions of all whisker base-points were projected onto the mystacial ellipsoid surface, and converted to spherical coordinates relative to the center ***c*** and axes of the mystacial ellipsoid. Thus, each *(x,y,z)* base-point was converted to (*r_BP_*, *θ_BP_,*, *ϕ_BP_*) shown in Figure 3A. This ensures that the base-points will move with the ellipsoid surface as the mystacial pad curvature changes.

As expected, significant linear relationships were observed between the *θ_BP_* angle and whisker column, and between the *ϕ_BP_* angle and the whisker row (p<0.001, two-way ANOVA). These relationships are illustrated in Figure 3B and 3C, and emerge because the whiskers are approximately aligned in a grid.

The average Cartesian distance between base-points was 1.8±0.05 mm for whiskers that were adjacent in a either a row or in a column. There were no significant correlations between whisker row and interwhisker distance (p = 0.52, two-way ANOVA). In contrast, there was a small negative correlation between whisker column and the interwhisker distance (p = 0.013, two-way ANOVA). This finding indicates that whisker rows converge closer to the nose whereas columns do not: the whiskers are spaced closer together in the dorsoventral direction, but maintain their average separation in the rostrocaudal direction.

#### Two-dimensional whisker shape: The first ∼50% of a whisker is approximately planar

A key assumption underlying the parameterization of whisker shape is that a significant fraction of the whisker's length lies in a single plane. A previous study, based on 105 whiskers, reported that the proximal-most 70% of a whisker is approximately planar [10]. In the present study, sufficient 3D and 2D data were available to validate this assumption for 84 whiskers (see Materials and Methods for exclusion criteria).

**Figure 4:**
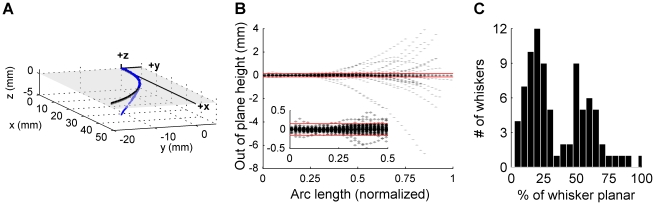
Out of plane curvature. (**A**) Comparison between the 3D scan (blue) and the 2D scan (black) for one whisker. The *xy*-plane is shaded and transparent to show 3D points above and below the plane. (**B**) Planar residuals for 84 whiskers. Individual whiskers are plotted in transparent gray, so that overlapping whisker points appear darker. Red lines indicate the planar threshold criteria of ±150 microns. Note that the whisker lengths are normalized by the 2D whisker lengths. Thus not all 3D whiskers extend to one (100%) because the 3D laser scanner did not always capture the entire length of the whisker (see Materials and Methods). Inset: Zoomed-in region from 0% to 50% of the normalized whisker length. (**C**) Histogram of the number of whiskers at each planar percentage. Bin size is 5%.

To determine planar residuals, 2D- and 3D-whisker scans were placed in a standard position and orientation (Figure 4A), defined by four criteria. First, the whisker base was defined to lie at the origin. Second, the initial linear portion of the whisker was forced to be collinear with the *x*-axis. Third, the planar portion of the whisker was set to be coplanar with the *xy*-plane. Finally, the whisker curvature was oriented to be negative, defined as a clockwise rotation between sequential segments when moving from base to tip (i.e. whisker concavity faces in the negative *y*-direction as shown in Figure 4A). In this orientation, the *z*-coordinate of every point along the 3D whisker is exactly identical to the planar residual at that point.

Figure 4B plots the planar residuals against whisker length normalized between zero and one, and illustrates that the majority of whiskers remain planar for approximately 50% of their length. The figure also demonstrates that the out-of-plane curvature increases closer to the tip. In general, the residuals are relatively small compared to the typical length of a whisker. The maximum deviation from the plane was found to be 6.8 mm, for a whisker of length 50.5 mm, resulting in the maximum ratio of out-of-plane curvature to total whisker length of 13.5% ( = 6.8/50.5). This whisker was an outlier – across all whiskers, the median out of plane curvature was 0.04 mm; the median ratio of out-of-plane curvature to total whisker length was 0.1%.

Using a strict planar threshold of 150 microns (red lines in Figure 4B), we found that the percentage of the whisker that was planar was described by a bimodal distribution. About half the whiskers were approximately 23% planar while the other half were approximately 63% planar (Figure 4C). A more liberal threshold will increase the percentage of the whisker that is considered planar, and in fact eliminates the bimodal distribution. The distribution becomes normal for a threshold of 300 microns (p>0.05, K-S test). For this threshold, 65% of the whisker is planar on average, in agreement with [10].

#### Two-dimensional whisker shape: Whisker length depends on both column and row

**Figure 5:**
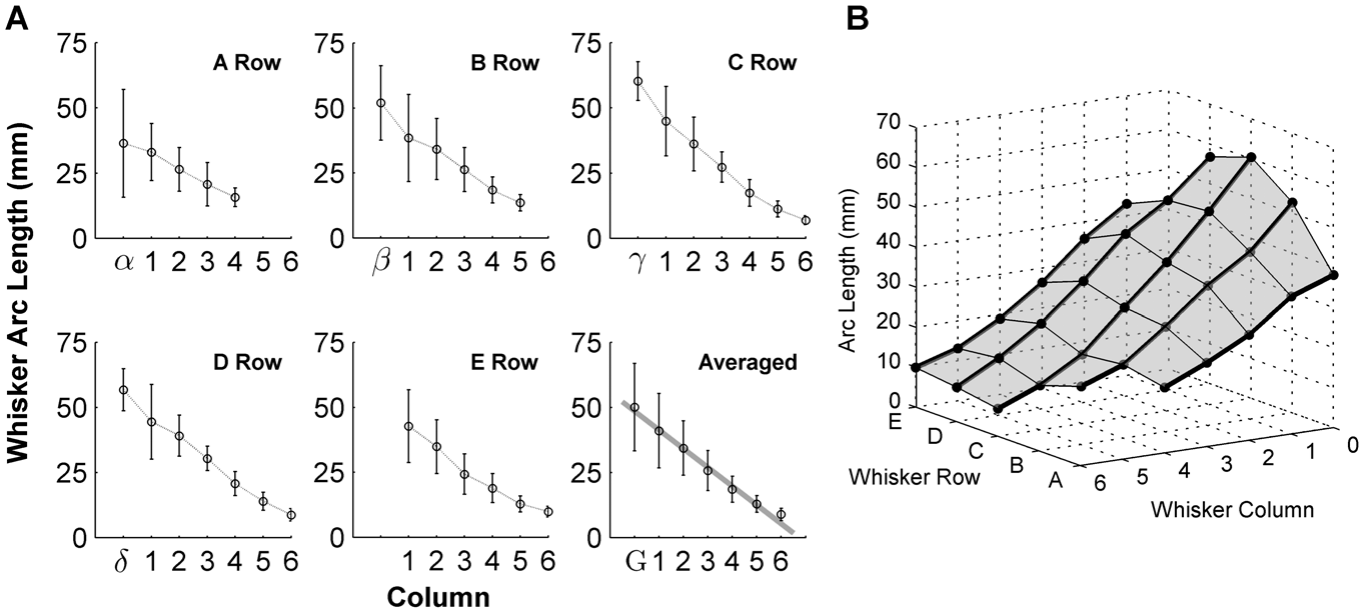
Whisker length dependence on position in the array. (**A**) Each panel shows whisker length as a function of column position within a single whisker row. Error bars are standard deviations of the mean in all panels (G = Greek). The solid gray line in the bottom right panel shows the best linear fit to the averaged data (correlation coefficient *r* = −0.74). (**B**) Thick black lines show the whisker length trend within a row (across columns) and the thin black lines show the trends within a column. Grey panels are visual aids only.

Previous studies have shown that the lengths of the macrovibrissae exhibit a strong dependence on position within the whisker array [11], [12]. One study [11], based on data collected from 15 rats, reported an exponential relationship between whisker length and column. The second study [12] used both adult (n = 8) and young (n = 7, <2 weeks-old) rats and showed a linear relationship. Our data, obtained from six adult rats and 354 whiskers, demonstrated that both linear and exponential fits were statistically significant (linear fit: r^2^ = 0.614; exponential fit: r^2^ = 0.606), and that there was no significant difference between the two types of fits (p = 0.87, F-test between two correlation coefficients). These results are shown in Figure 5A.

The whisker length was influenced significantly by both the whisker row and the column (p<0.001, two-way ANOVA). The surface plot of Figure 5B provides an intuition for how the whisker lengths vary across the array. For example, it illustrates that the Greek whiskers are the longest within a given row, and that the C and D row whiskers are generally longer than the A, B or E row whiskers.

#### Two-dimensional whisker shape: Whiskers are shaped approximately as parabolas whose intrinsic curvature depends only on column, not on row

**Figure 6:**
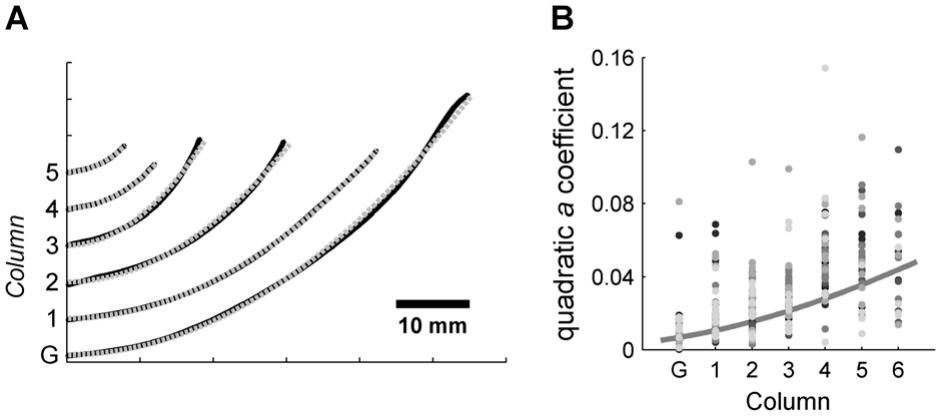
Quadratic approximation to the whisker 2D shape. (**A**) Examples of quadratic fits to the right C row whiskers from one rat. The scanned whisker data is shown in black. Each whisker has been offset vertically to reflect the whisker's column. The best parabolic fit to each whisker is shown as a dotted gray line. (**B**) Quadratic coefficient *a* versus whisker column in the array for all 2D whisker data. Marker color indicates rat of origin and indicates that no significant differences were found across rats.

Preliminary analysis confirmed the results of an earlier study demonstrating that the 2D shape of the whisker is well-approximated by a parabola [10]. Although a parabolic fit is not a coordinate-free representation, it is an intuitive fit compared to alternatives such as Césaro coefficients (described in Supplementary Information Text S1, Table S1 and Figure S1). A general parabolic fit to the whisker would take the form 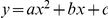. However, to more easily compare across whiskers, we fit the whiskers to a quadratic curve with only one coefficient: 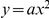. This single parameter (*a* coefficient) faithfully captured the shape of the whisker (statistics listed in Materials and Methods). Examples of quadratic fits to whiskers from the C row of a single rat are shown in Figure 6A.

A two-way ANOVA was performed between the quadratic coefficient *a* and whisker row and whisker column. A significant relationship was found for whisker column (p<0.001), but not for whisker row (p = 0.02). The model that best described the relationship between the coefficient *a* and whisker column was exponential. The exponential model resulted in a fit (r^2^ = 0.37, data shown in Figure 6B) that was significantly stronger than a linear relationship (r^2^ = 0.29). The strong correlation between quadratic coefficient and whisker column necessarily implies a strong correlation between the quadratic coefficient and whisker length.

#### Three-dimensional whisker orientation: The angles at which the whiskers emerge from the mystacial pad depend on row and column

The 3D orientation of the whiskers was defined in terms of projection angles instead of Euler angles because projection angles have a clear physical meaning in behavioral studies. Supplementary Information Text S1 provides the relationship between the projection angles and Euler angles, which are likely to be useful in future modeling studies.

**Figure 7:**
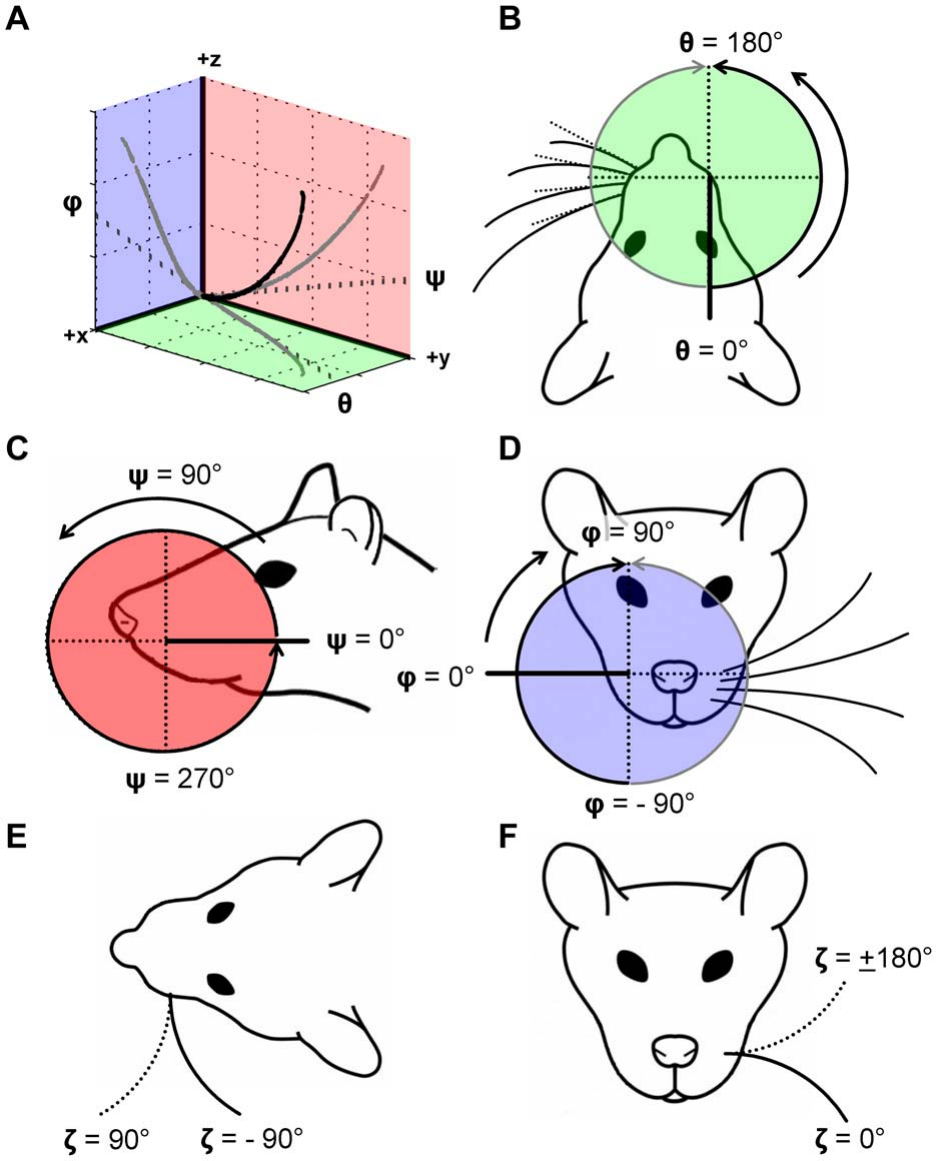
Whisker angle convention on the rat head. (**A**) Schematic of projection angles that describe whisker orientation in 3D. Whisker is indicated by the thick black line. Whisker projections into the three planes of the head coordinate frame are shown in gray, along with the corresponding projection angle. (**B**) Definition and range of the angle *θ*. *θ* increases from 0° to 180° on both right and left sides as the whisker protracts (identical definition for *θ* as [13], [14]). (**C**) Definition and range of the angle *ψ*. (**D**) Definition and range of the angle *ϕ*. (**E, F**) Definition and range of the angle *ζ* (identical definition for *ζ* as [10]). Whiskers on the right side of the face follow the same convention as the left for *ζ*; therefore, a *ζ* of +90° has the whisker tip pointing forward for both the left and right side of the face.

Three whisker projection angles (*θ, ϕ, ψ*) were computed by projecting the linear portion of the 3D whisker onto each of the three Cartesian planes (*xy*, *xz*, and *yz*). Projection angle conventions are shown in Figure 7 and are based on the standard orientation of the head (Figure 1). It can be more intuitive to think of the three planes in terms of anatomy: the *xy*-plane is equivalent to the horizontal plane (containing *θ*, Figure 7B, identical to [13], [14]); the *yz*-plane is equivalent to the sagittal plane (containing *ψ*, Figure 7C); and the *xz*-plane is equivalent to the coronal plane (containing *ϕ*, Figure 7D).

Because rat whiskers have an intrinsic curvature, a fourth variable is required to orient the whisker. This angle of rotation about the whisker's own axis has been defined previously in the literature as *ζ* [10] and is shown in Figure 7E and 7F.

**Figure 8:**
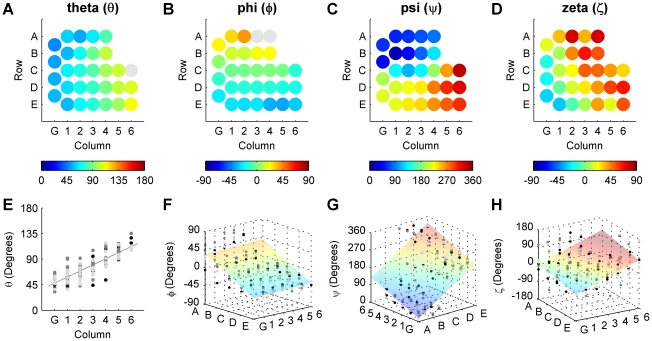
Dependence of *θ*, *ϕ*, *ψ* and *ζ* on whisker identity (row and column location in the array). (**A–D**) The angles *θ*, *ϕ*, *ψ* and *ζ* for each whisker, averaged across both right and left sides of all three rats. Color scales are different for each subplot. (**E–H**) The angles *θ*, *ϕ*, *ψ* and *ζ* obtained from all rats, plotted against the dominant factor(s) (either row or column or both). Different marker colors (black, gray, and light gray) indicate rat of origin, and indicate that no significant differences were found across rats.

Average orientation angles obtained for the left and right arrays are shown in Figures 8A–8D (for Euler angles see Supplementary Information Text S1 and Figure S2). Whisker identity is shown in a simplified matrix arrangement, and the magnitude of each whisker angle is denoted by color. These figures clearly show that the orientation angles vary smoothly across the array. The orientation angle *θ* varies most strongly with column, with little (but significant) variation as a function of row. In contrast, *ϕ* varies most strongly with row and varies little with column. The remaining two angles (*ψ* and *ζ*) depend on both row identity and column identity. This dual dependence is visible in Figure 8C and 8D as a gradation in color within each specific row and column. It is particularly evident in the plot of *ψ*, in which the colors vary smoothly in a diagonal pattern.

A two-way ANOVA (with row and column as the independent factors) demonstrated that each orientation angle depended on both column and row (p<0.001). However, many of these dependencies indicated that a single row or column deviated significantly from the mean. For example, the average theta angle in the A row was 10° more retracted on average than all other row theta angles. In general, although both row and column are significant factors, one factor typically dominated the trend. In other words, the p-value for one factor was always much smaller in magnitude than the p-value for the other factor. The dominating factors were column for *θ*, row for *ϕ*, row for *ψ* and column for *ζ*. The raw data and resulting trend for each orientation angle are shown in Figures 8E–8H.

### The model vibrissal array and its equations

#### Equations relating each whisker-specific parameter to whisker identity

The final equations relating each of the eight whisker parameters (*θ_bp_, ϕ_bp_, s, a, θ, ϕ, ψ, ζ*) and whisker row-column identity are summarized in Table 3. As described in Materials and Methods, four different types of relationships were tested, and to avoid over-fitting, a higher-degree or non-polynomial fit was chosen as the appropriate fit only if it was significantly better than the linear fit (F-test of the correlation coefficients, p<0.001).

**Table 3:** Equations relating each whisker parameter to whisker identity.

Parameter		Dependencies	Type of Equation	Equation	R^2^
Base point Theta	θ_BP_	Column	Linear	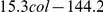	0.72
Base point Phi	ϕ_BP_	Row	Linear	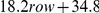	0.65
Arc length	s	Row, Column	2D Linear	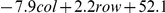	0.64
Quadratic Coefficient	a	Column	Exponential	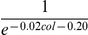	0.37
Theta	θ	Column	Linear	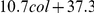	0.71
Phi	ϕ	Row, Column	2D Linear	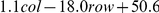	0.82
Psi	ψ	Row, Column	2D Linear	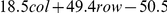	0.66
Zeta	ζ	Row, Column	2D Linear	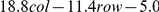	0.47

#### A single equation that combines all parameters to generate the full macrovibrissal array

The final step in generating the model vibrissal array was to combine the seven mystacial pad parameters (Table 2) and eight whisker parameters (Table 3) in a purely geometrical model. Because the parameters are related geometrically, a single equation can be constructed that describes the location of every point on every whisker in the array. The equation was constructed by first defining the 2D whisker equation, and then applying the 3D rotation and translation required to put this 2D whisker in the appropriate position in the 3D array.

Let *w_2D_* be the (*x,y,z*) coordinate of a point on the 2D whisker curve. In our parameterization,
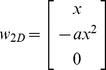
(1)where *x* is a function of both arc length, *s*, and the parabolic shape coefficient, *a*, written as
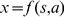
(2)The function *f(s,a)* is nontrivial, and is discussed in Supplementary Information Text S1.

To place this point in its appropriate 3D position, it first must be rotated by the Euler angles (*θ_e_, ϕ_e_, and ζ_e_*) specific to this particular whisker, and then translated by an amount equal to its base-point location on the ellipsoid.

To rotate the point requires conversion between projection angles and Euler angles, which is straightforward and described in Supplementary Information Text S1. In equation form, this becomes:
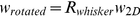
(3)where *w* is an arbitrary point along a whisker, and
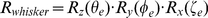
(4)In equation 4, *R_z_, R_y_, and R_x_* are standard rotation matrices that describe rotations about the *z*-, *y*-, and *x*-axes, After the point is rotated, it is then translated (offset) by an amount equal to the whisker's 3D base position, *w_3D_*, yielding:
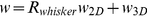
(5)Because the whisker base location *w_3D_* was represented in terms of the mystacial ellipsoid, it can be expressed as:
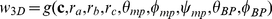
(6)where ***c*** is the (*x,y,z*) ellipsoid center location, (*r_a_, r_b_, r_c_*) are the ellipsoid radii along the major axis, semi-major axis and minor axis, respectively, (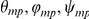) are the ellipsoid orientation angles specified in Euler angles, and (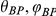) is the base-point location in spherical coordinates relative to the ellipsoid axes. The variable *r_BP_* does not appear in the expression for *w_3D_* because the whisker base-point is constrained to lie on the ellipsoid surface. The actual function (g) for *w_3D_* is described as:
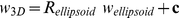
(7)where
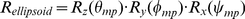
(8)

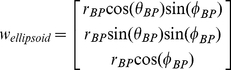
(9)and
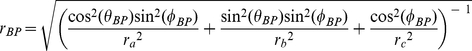
(10)The final equation for any arbitrary point, *w*, within the model whisker array exploits the feature that every whisker parameter depends on row and/or column and the ellipsoid parameters depend on the side of the face to which the whisker belongs. The final equation thus simplifies to:
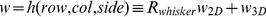
(11)


#### The final model of the array captures essential features of the vibrissal array

**Figure 9:**
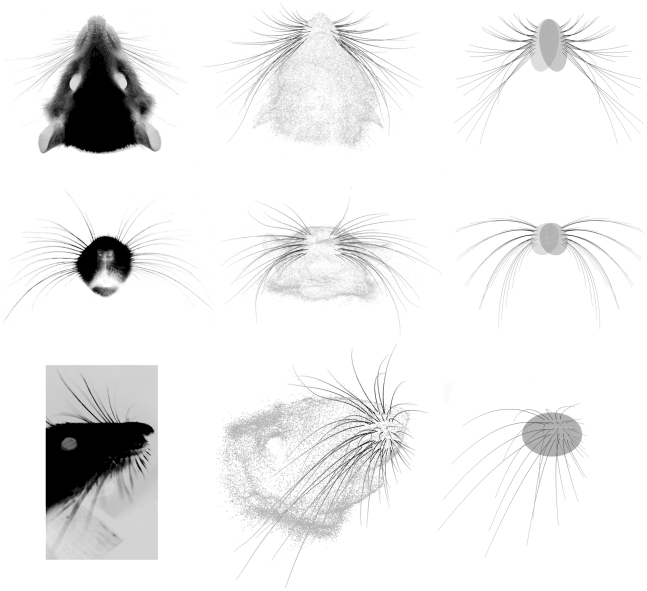
Comparison between photographs of the vibrissal array, 3D scans of the vibrissal array, and the model of the vibrissal array. Care was taken to ensure similar head orientations for all images within a row. (**Left column**) Photographs of an anesthetized rat. (**Middle column**) Scanned 3D images of another rat. (**Right column**) Model of the vibrissal array generated from the parameter values in Table 2, equations in Table 3, and equation 10.

The final model of the rat vibrissal array generated using the equations from Table 3 and equation 11 is shown in the right column of Figure 9. The Matlab functions written to generate the final model are included in the Supplementary Information Dataset S1. For comparison, one of the 3D scanned rats is shown in the middle column, and photographs of an anesthetized rat (not used for any of the 2D or the 3D scans) are presented in the left column. A visual comparison of the photographs and the 3D scans provides a sense of the variability across individuals and our scanner resolution. It also provides visual confirmation that differences between the model array and either the photograph or the scan are on the same order as differences between the photograph or the scan.

### Error analysis of the model

To evaluate the accuracy and precision of the model, an uncertainty analysis was performed. We used error propagation techniques to compute the variance of any point on the array given the variances of each of the model parameters [15]. To perform this propagation of error analysis, we determined the variability in each parameter across animals, and then examined how that variability would propagate through the final equation (eq. 11) used to generate the model. This analysis gives the variance of the model.

Uncertainty in the model was determined by quantifying how variance in each of the parameters (*θ_mp_, ϕ_mp_, ψ_mp_, θ_bp_ ϕ_bp_, s*, and *a*) affected the location of every point along every whisker in the model. Note that orientation angles are not appropriate parameters to include in the error propagation analysis because these angles are actively controlled by the rat during whisking behavior and may be considered “inputs” to the model (see Discussion).

**Figure 10:**
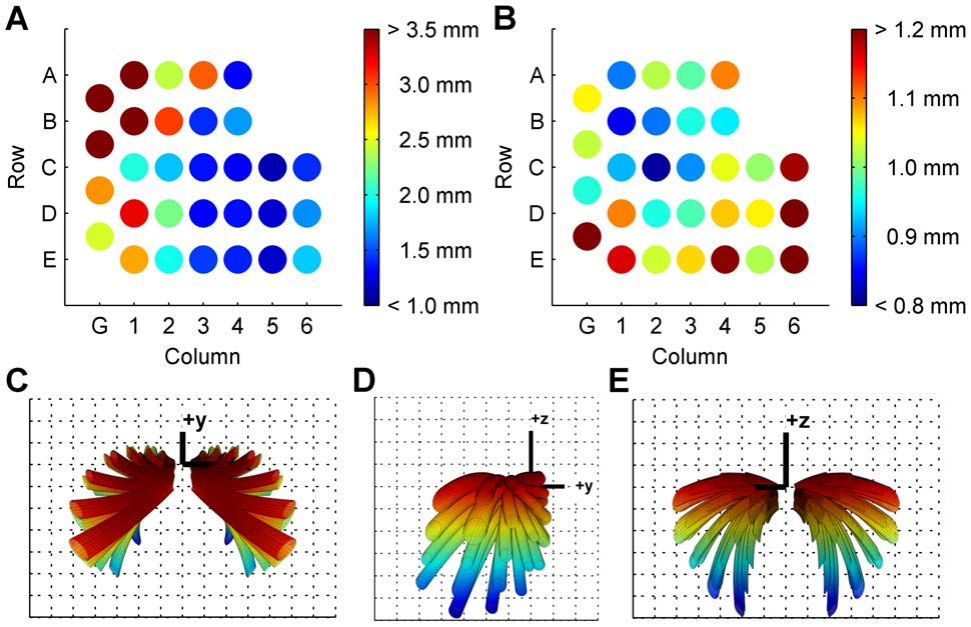
Error in whisker position. (**A**) Standard error (mm) at each whisker tip. (**B**) Standard error (mm) at each whisker base. (**C–E**) Horizontal, sagittal, and coronal views of the final model with error surfaces surrounding each whisker. The error cylinder radius at each arc length is equal to the 95% confidence interval based on the propagated standard deviation and assuming a normal distribution of errors. Colors in C–E are visual aids only; they do not represent error.

Results of the error analysis for the locations of the whisker tip and base-points are shown in Figure 10. The tips of the whiskers are particularly useful locations to quantify error for two reasons: first, the tips were not explicitly modeled, and second, the tips are the most distal part of the array, and consequently will have the largest error of any point along the whisker. The whisker tip positions thus serve as the most conservative upper bound on the error in the model. Across all whiskers, the average standard error in tip position was 3.2±1.7 mm (standard deviation: 7.8±4.2 mm), and is depicted graphically in Figure 10A. The error in tip position was computed as the Euclidean distance between the location of the whisker tip and the tip point plus the standard deviation of the tip's *(x, y, z)* coordinates.

Across all whiskers, the average standard error at the whisker base was 1.3±0.26 mm (standard deviation 3.7±0.65 mm), and is depicted graphically in Figure 10B. The error in base position was computed as for tip positions. The 95% confidence interval for each point along the whisker arc length is shown graphically in Figure 10C, 10D, and 10E using colored surfaces. A conservative estimate of maximum error in our model is that tip positions are accurate to within a standard error of ±5 mm (3.2+1.7 mm).

To generalize across whiskers, error was also quantified as a function of arc length. The standard error of any point along any whisker divided by the arc length between that point and the whisker base was typically 5%±1% of the arc length. This means, for example, that a point at an arc length of 10 mm will have a standard error of 0.5 mm.

### Embodied sensing: How the morphology of the whisker array affects patterns of input

Simulations using the model of the vibrissal array can provide insight into the patterns of mechanosensory input that might be generated as the rat actively explores an object. The morphology of the array directly affects how the whiskers contact the object, and thus the mechanical information available to the nervous system. This information in turn constrains the set of neural computations that can be used to extract particular object features. Here we consider – under a particular set of assumptions about head and whisker movement – how the morphology of the vibrissal array constrains the type of neural computations that could enable the rat to determine the curvature of an object.

#### Whisker-object contact patterns provide information that can be used to compute a cylinder's curvature

Investigating the relationship between the morphology of the vibrissal array and whisker-object contact patterns was predicated on several assumptions about head and whisker movement. The three most important assumptions were: (1) the head was stationary over the course of a protraction (slightly greater than ½ of the whisking cycle), (2) the whiskers within a row rotate in a single plane, and (3) the whiskers do not rotate about their own axis (the angle *ζ* stays constant). The results presented here must be interpreted in light of these three assumptions. The results presented here do not make any assumptions about whisking velocity.

**Figure 11:**
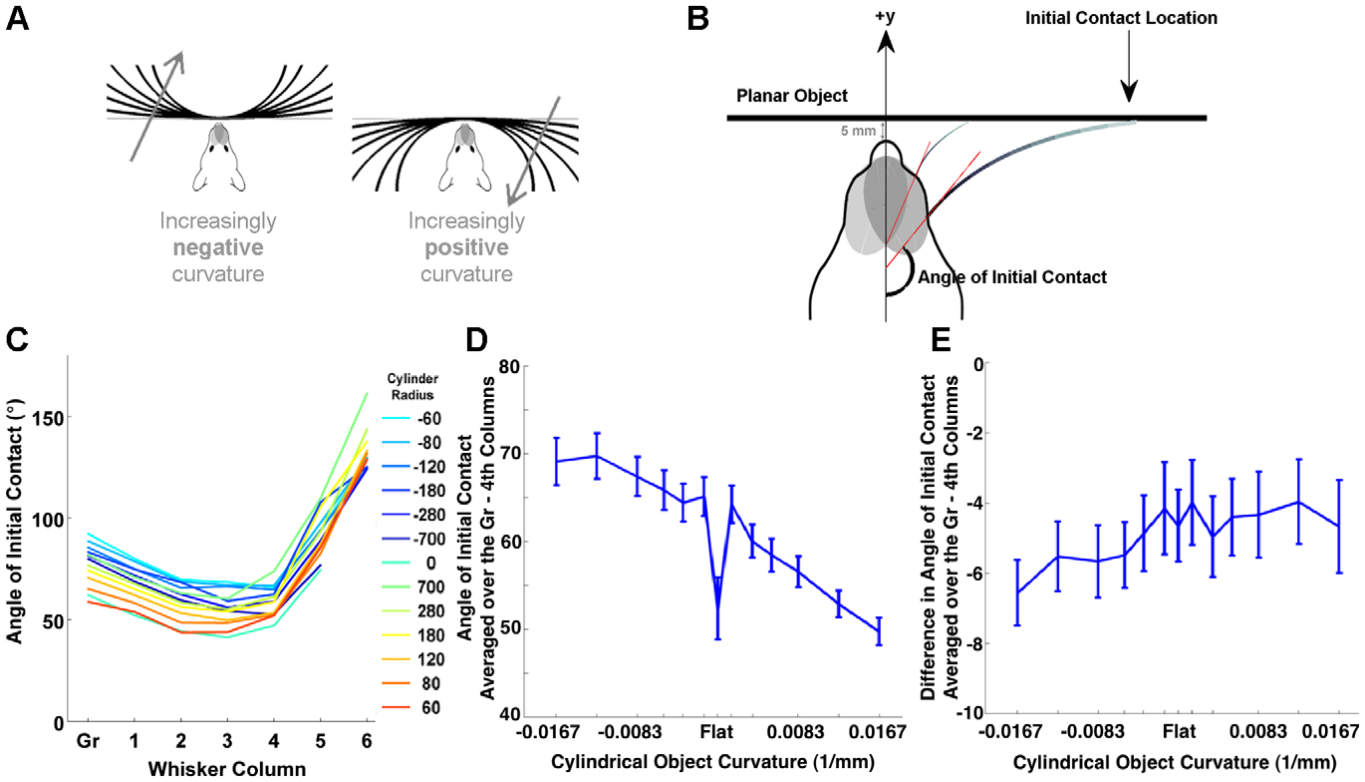
Array morphology constrains the information available to the nervous system about object curvature. (**A**) Schematic showing the different cylinder radii tested. Negative radii and curvature were defined such that the convex face of the object faced the rat. Note that the cylinder approaches a plane as the radius goes to infinity and curvature goes to zero. (**B**) Schematic illustrating the calculation of the angle of initial contact. Red dashed line indicates the angle at the base of the whisker at which the whisker makes its first contact with the object. This figure also illustrates a situation in which a more rostral whisker will contact at a more protracted angle than a more caudal whisker. (**C**) Angles of initial contact are shown for each whisker of the C row. Each trace represents results for a cylinder with a different radius, color coded as shown in the legend. *Gr* indicates the gamma whisker. (**D**) Average angle of initial contact versus cylinder curvature (1/radius). The most curved cylinders are represented at the graph extremes. (**E**) Difference across columns in the average angles of initial contact versus cylinder curvature. In both D and E, error bars indicate the standard error of the mean (averaged across all whiskers in the model).

As shown in Figure 11A, virtual cylinders of different radii were placed symmetrically 5 mm in front of the model of the vibrissal array. Cylinders had radii of ±60, 80, 120, 180, 280, 700 mm. A flat wall (cylinder of infinite radius) was also tested. Whiskers were simulated to rotate in a plane until they collided with the cylinder. The angle at which each whisker first contacted the cylinder was defined as the “angle of initial contact” and is schematized for two whiskers in Figure 11B for the case of the flat wall. Details on how the simulations were performed and how the plane of rotation was chosen are provided in Materials and Methods.

Figure 11C plots the angle of initial contact for whiskers in the C row as a function of whisker column, for different values of the cylinder radius. Data for the other whisker rows followed the same trends as data for the C-row, but are not included in the plot to ensure visual clarity. The curves of Figure 11C demonstrate that the middle columns of the whisker array (columns 2–4) tend to hit the object at a more retracted angle than their caudal or rostral counterparts. Surprisingly, whiskers in the most rostral columns (columns 5–6) hit at more protracted angles than the other columns. This was true even though the object was placed at a distance smaller than the length of the most-rostral whiskers (the smallest whisker arc length is 5.23 mm). This effect occurs because the whiskers have an intrinsic curvature. Figure 11B shows an example in which a smaller, more rostral whisker will contact at a more protracted angle than a longer, more caudal whisker.

It is clear that each of the curves in Figure 11C is offset by a value related to the radius of the cylinder, and therefore, that the angle of initial contact averaged across columns could be used to determine the curvature of the object. Figure 11D plots the angle of initial contact averaged across all rows and the first five columns of the whisker array (Greek-4^th^) versus the cylinder curvature (1/radius). The relationship is roughly linear at non-zero curvatures, with the initial angle of contact proportional to the curvature. Neurons in the rat's brain could potentially learn this linear relationship to allow the rat to determine the curvature of an object within the time scale of a single protraction.

An alternative strategy for calculating the cylinder's curvature might be to use information present in the difference between the angles of initial contact across neighboring columns. To compute this measure, the initial contact angles were averaged across all rows within a column. The difference of this average was then calculated between the first five neighboring columns of the array (Greek-4^th^). Figure 11E plots this difference across columns as a function of cylinder curvature. The figure clearly demonstrates that this measure is not uniquely related to cylinder curvature. Neurons in the rat's brain could not make sole use of information present in difference between the angles of initial contact across neighboring columns to determine the curvature of an object.

These results demonstrate that array morphology directly constrains the relationship between the mechanical signals generated during whisking behavior and the information available to the nervous system about particular object features. To obtain a unique determination of object curvature, the nervous system could use a computational strategy based on the absolute angles of initial contact, but could not rely solely on a strategy based on the difference in angles of initial contact. We next asked to what degree changes in array morphology would change these constraints on neural computation.

#### The computational strategy used to compute curvature depends critically on the array morphology

Two alternate array morphologies were tested in the same simulations of whisker-object contact. The array morphology was altered by a single parameter: the angle *ζ*, which describes the rotation of the whisker about its own axis, was set either to +90 degrees or −90 degrees for all whiskers. As shown in Figure 7, a value of *ζ* equal to +90 degrees means that all whiskers will be oriented concave forward, while a value of *ζ* equal to −90 degrees means that all whiskers will be oriented concave backward.

**Figure 12:**
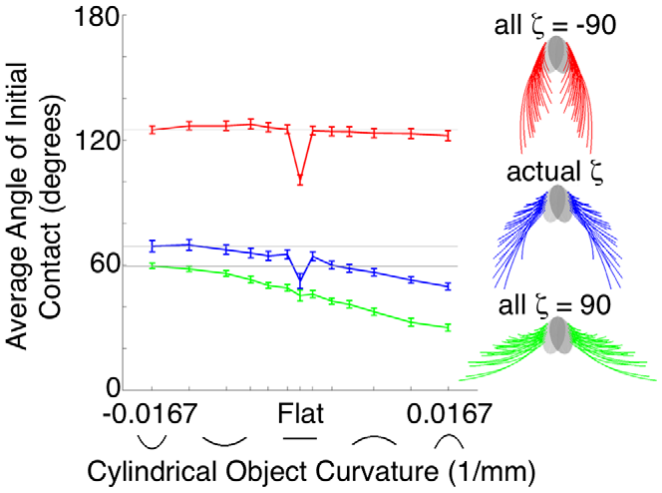
Effect of array morphology on information available to the nervous system. Average angles of initial contact with cylinders of varying curvatures were calculated for each array morphology. If all whiskers are oriented concave forward (ζ = +90, green), the relationship between the average angle of contact and the object curvature is mostly a shifted version of the relationship obtained for the actual morphology (blue). If all whiskers are oriented concave backwards (ζ = −90, red), the functional relationship changes dramatically. No significant relationship exists between the angle of initial contact and object curvature. Gray lines show the value of the mean angle of contact for the largest negative curvature for each morphology, and are intended to guide the eye.

Figure 12 compares the results of simulations with the alternate array morphologies with results from the simulation with the actual array morphology. The figure demonstrates that if all whiskers were oriented concave forward (*ζ* = +90), the relationship between the average angle of contact and the object curvature is merely a shifted version of the relationship obtained for the actual morphology. Neurons in the rat's brain could still potentially learn a linear relationship to relate the average angle of contact and the object radius.

In contrast, if all whiskers are oriented concave backwards (*ζ* = −90), the relationship between the angle of initial contact and the object curvature changes dramatically. In fact, no significant relationship exists between the angle of initial contact and object curvature. The nervous system would need to employ a different computational mechanism to determine object curvature.

## Discussion

In the field of motor systems, it is generally recognized that control is shared between the nervous system and the periphery [16], [17]. Animal movement depends on the passive dynamics of the limbs and muscle biomechanics as well as on descending motor commands and sensory feedback.

Similarly, sensory data acquired by the nervous system is constrained by the embodiment of the peripheral sensory organ: its material, its morphology, and its mechanics. The embodiment of the sensory organ shapes the physical signals that can be gathered and transmitted to receptors and ultimately to the brain. Thus, the neural circuits that subserve sensing and perception must evolve in tandem with the physical embodiment of sensory structures.

The rat vibrissal array is a particularly good model system in which to study the intertwined nature of embodiment and neural processing during sensory acquisition behaviors. Tactile input from the perioral regions subserves a wide repertoire of rodent behaviors, ranging from ingestion [18] to social and aggressive behavior [19] to shape and texture discrimination [4], [5], [6], [7], [8], yet the whisker array has a relatively simple structure compared to that of the hand. The whiskers have a highly regular spatial arrangement, movements of the vibrissal array are largely rhythmic, and the whiskers cannot manipulate or grasp objects. The rich set of behaviors that rely on vibrissal tactile input, coupled with the array's relatively stereotyped morphology and biomechanics, permits systematic examination of how alterations in morphology differentially affect aspects of sensing behaviors (e.g., the extraction of object curvature, as in the present study).

### Embodiment of the rat vibrissal array

Embodiment of the vibrissal array can be conceptualized as spanning at least four levels: the material properties of a single whisker (e.g. elasticity and damping), the morphology of a single whisker, the musculature and tissue surrounding each whisker, and the morphology of the entire whisker array. The present study examines this fourth level.

Previous studies of vibrissal array morphology qualitatively characterized the locations of whisker base-points and identified systematic variations in whisker length [11], [12]. The grid-like arrangement of the vibrissae is one of the most easily observable features of mystical pad anatomy [1] [3]. Brecht et al. demonstrated that a set of governing geometric principles, conserved across species, could *qualitatively* explain this grid-like arrangement [11].

The present study now *quantifies* the three-dimensional vibrissal array architecture. Specifically, we provide a single equation that describes every point of every whisker. This work adds to the understanding of vibrissal array morphology in several important ways. First, the locations of the whisker base-points are quantified in three-dimensions, capturing the strong row- and column-based structure of the array, while also incorporating the underlying curve of the rat's mystacial pad. Second, equations define the 2D shape of each whisker in terms of its intrinsic curvature as well as its length, and the angle *ζ* describes the orientation of each whisker's intrinsic curvature. The orientation of a whisker during protraction will affect both the angle and time at which it will make contact with an object, and a recent study has shown that the rat can change *ζ* during a protraction [10]. Thus the intrinsic curvature as well as *ζ* are essential parameters for accurate models of whisker movement. Finally, quantifying vibrissal array features in analytical form allows for systematic, cross-species comparisons of structure-function relationships in the context of behavior and ethological niche, as described below.

### Morphometric analysis and cross-species comparisons

The present study uses basic techniques from geometric morphometrics to analyze the morphology of the rat vibrissal array. In general terms, morphometrics refers to the quantification of variations in the shapes of objects. When applied to biology, morphometrics can be used to quantify and compare the shapes of organisms within and across species [20]. In some “landmark-based” analyses, for example, deviations of individual specimens from an average morphology have revealed subtle morphological differences between taxa, sexes, ages, and geographic locations [21].

Importantly, morphometric analysis has also been used to distinguish morphological differences attributable to phylogeny from those arising from behaviors that a species or sub-species has adopted in response to the local environment. Once morphological features have been quantified, statistical techniques can provide estimates of which morphological variations are best explained by phylogenetic differences and which by environmental factors (for an example, see [22]).

The model developed in the present study lays the groundwork to investigate the origin of cross-species differences in the morphology of the vibrissal array. For example, murid rodents closely related to the rat (e.g., the mouse) may have a vibrissal morphology that simply scales with body size (a phylogenetic effect). More distant species may show changes in morphology that reflect adaptive traits enabling behaviors essential for species survival.

### Use of the model in kinematic simulations of whisking behavior

The orientation angles of the whiskers *(θ, ϕ, ψ, ζ)* are the angles at which the whiskers emerge from the rat's face. The facial muscles of the rat control the orientation angles, and these muscles have many degrees of freedom. They can move the whisker through a large range of orientation angles during natural whisking behavior. Because the 3D whisker scans were done post-mortem, rigor mortis of the facial muscles could have pulled the whiskers to orientation angles anywhere within their natural range of movement. Unsurprisingly, these angles were the parameters that exhibited the largest variability across animals, although smooth trends are clearly visible (Figure 8).

The variability associated with the static, post-rigor measurement of orientation angle is not particularly meaningful, as the muscles could have pulled the whiskers into any number of orientation angles. Accordingly, this variability was deliberately excluded from the error analysis of the model. The orientation angles should not be thought of as fixed parameters of the model, but should instead be used as inputs to the model to simulate whisking behavior.

Numerous studies have quantified changes in the horizontal angle *θ* during natural whisking [5], [7], [9], [10], [13], [14], [24], [25], [26], [27], [28], [29]. At least two studies have produced data from which it is possible to determine how the angles *ϕ* and *ψ* change over the course of a whisk [10], [25]. Finally, Knutsen et al. provided the first evidence that the whisker rolls about its own axis during active whisking. The roll can be described by the value of the angle *ζ* over the whisking cycle. Taken together, these studies determine the equations for changes in orientation angles throughout the whisking trajectory.

In addition to the equations that describe whisker movements in terms of changes in orientation angles, a complete model of whisking kinematics will require an equation that relates the protraction angle *θ* to mystacial pad parameters. The mystacial pad translates [9] and also changes shape during the whisk cycle (unpublished observation), causing the whisker base-points to translate. In the model, positions of the whisker base-points translate with the underlying mystacial pad [9], and therefore can be determined based solely on ellipsoid shape.

There are several potential uses of an accurate kinematic model of the vibrissal array; most importantly, it could be used to simulate the expected spatiotemporal patterns of whisker-object contact during exploratory behaviors. As shown in the present study, even without information about whisker velocity, the model can be used to simulate relationships between the sensory signals acquired and an object feature. However, these simulations only predicted the angles of whisker-object contact, while an accurate kinematic model could also predict the temporal sequence of contacts. Inter-whisker contact intervals are important in determining whether neural responses in barrel cortex will be suppressed or facilitated [23], [24], [25], [26]. A predictive model of kinematics could thus be used to probe expected neural responses given the head's position and orientation relative to an object.

A second possible use of the model is as a predictive tool to describe where whiskers *should* be. This could be especially helpful when tracking whiskers in behavioral datasets. In high speed videography, whiskers are often observed to cross, go out of focus, and blur. Predicting the 2D projection of a whisker could reduce the search space for a whisker-tracking algorithm, and provide an estimate of whisker identity at the same time.

### Conclusions

A growing theme in sensory neuroscience is the need to link movement and sensing behaviors [27]. The present study reflects the increasing need for simulations of dynamics in sensory neuroscience, as the model represents a first step towards the creation of an entirely “digital rat,” that is, a simulation platform to test theories of whisker movement, mechanical modeling of whisker-object collisions, mechanotransduction, and neural coding.

The morphology of the vibrissal array directly constrains the mechanosensory inputs that will be generated during behavior. The morphological model can be used in conjunction with models of whisker kinematics or dynamics to develop increasingly accurate predictions about exploratory patterns of freely behaving animals, and thus the neural computations that can be associated with extraction of particular object features. Responses of sensory neurons are likely to be conditioned on, or tuned to, the particular movements used to extract the data.

An improved understanding of the dynamics of motor systems that acquire sensory data will improve our understanding of the complex interactions between sensorimotor structures, the nervous system, and the specific environments in which they function.

## Materials and Methods

### Ethics statement

Animal protocols for this study were written in strict accordance with the recommendations in the Guide for the Care and Use of Laboratory Animals of the National Institutes of Health, and were approved in advance by the Animal Care and Use Committee of Northwestern University.

### Specimens

A total of six adult, female Sprague-Dawley rats (>3 months old, approximately 300 grams) were used.

The head and vibrissal arrays of three of the six rats were scanned in a three dimensional (3D) volumetric scanner. Rats were euthanized with a lethal dose of pentobarbital. Tiny incisions (∼1 mm) were made in the scalp to accommodate the implantation of four skull screws. Rigid positioning rods were attached to the screws with dental acrylic. The rat was decapitated and the positioning rods were used to mount the head stably within the 3D scanner. Three-dimensional scans occurred within two hours of euthanasia (after rigor mortis had set in). Immediately after the 3D scan, each macrovibrissa was grasped firmly at its base with tweezers and plucked in a single swift motion from the mystacial pad. Each isolated macrovibrissa was then scanned in two dimensions (2D) on a flatbed scanner within 4–12 hours after euthanasia. The remaining three rats were euthanized in unrelated experiments, and did not undergo 3D scanning. Their macrovibrissae were scanned in 2D, again within 4–12 hours after euthanasia. Neither the animals nor the whiskers were preserved prior to scanning in any way.

Scans of whiskers in the A, B, C, D and E rows, along with the Greek column α, β, γ and δ, were obtained from both right and left whisker arrays. The first six whiskers of each row were analyzed because these whiskers are associated with sling muscles [3] and are therefore considered macrovibrissae. The arrangement of the microvibrissae was not quantified. In our specimens, the A row contained only four whiskers. The B row consistently contained four whiskers on the left side and five whiskers on the right side; this peculiarity is perhaps due to the specific strain of rats that used in the study (Sprague-Dawley). The C, D and E rows all contained six or more whiskers. This arrangement of whiskers is consistent with that described in previous reports [3], [11].

### Three dimensional volumetric scan and data extraction

**Figure 13:**
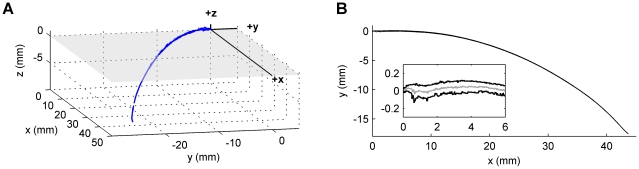
Experimental dataset and standard orientations. (**A**) An example whisker (right B1), extracted in 3D from the scanned rat head shown in Figure 1A of Results. (**B**) Shape of the whisker extracted from the 2D scan. The inset shows a magnified portion of the whisker with both x and y axes in units of mm. The light gray line is the centerline.

A Surveyor DS-3040 3D laser scanner (Laser Design Incorporated) was used for the 3D scans. The final scanner output was a finely digitized 3D point cloud (20 micron volumetric accuracy) as shown in Figure 1A of Results. The complete point cloud – including both head and whiskers – was imported into the software package RAPIDFORM XOR. Within this software package, data points corresponding to each macrovibrissae were extracted from the point cloud. An example of an extracted whisker is shown in Figure 13A. The extraction process involved manual rotation of the 3D scanned image to visually determine the set of points that clearly belonged to each whisker through all angles of rotation. The whisker base-point was then identified as the centroid of a small number of points (typically 8–20) on the mystacial pad that rotated the least relative to that identified macrovibrissa. Whisker identity was assigned using the well-known topographic arrangement of whiskers on the mystacial pad [3]. In the case that two or more whiskers emerged from the same follicle (as occurred for approximately 10% of follicles), only the largest whisker was used. After manual extraction, the 3D points belonging to each macrovibrissa were exported to Matlab and a moving average (21-sample window) was used to smooth the shape.

### Two dimensional scan and data extraction

The macrovibrissae from all six rats were plucked and scanned in 2D using a flatbed scanner. Isolated vibrissae were scanned at spatial resolutions ranging from ∼10.6 microns/pixel to ∼3 microns/pixel using either an Epson Perfection 4180 or a UMAX Powerlook 2100 XL scanner. Two scanners were used to quickly and efficiently image the large number of whiskers.

The 2D whisker shape was extracted using custom image processing algorithms. Each image was converted to black and white either in Adobe Photoshop v.7 or in Matlab, and the whisker outline was extracted in Matlab (Figure 13B). When magnified, the upper and the lower edges of the whisker became apparent (heavy black lines in the inset of Figure 13B). The midpoint between the upper and lower whisker edges was determined at small increments along the extracted whisker length. The midpoints were connected to obtain the centerline of the whisker, which closely matched the overall whisker shape (grey line in the inset of Figure 13B). Throughout Results, 2D whisker shape was quantified using the centerline of the whisker.

### Parameterization of the vibrissal array

The vibrissal array was parameterized in terms of variables that are relatively easy to measure in behavioral studies. This parameterization relies on seven parameters specific to the mystacial pad and eight parameters specific to each whisker. These 15 parameters are listed in Table 1 of Results and described in more detail below:

#### Mystacial pad: position, shape, and orientation (c, r_a_, r_b_, r_c_, θ_mp_, ϕ_mp_, ψ_mp_)

Four different surfaces were evaluated as candidate models for the mystacial pad: sphere, cone, cylinder and ellipsoid. First, the base-points of all of the whiskers on both sides of the face (described in *x,y,z* coordinates) were scaled such that the norm of the distance matrix was the same for all rats. The distance matrix contains the distance between every pair of points, including points on both right and left sides. Second, each surface was fit to the normalized base-points of each side of the face separately using least-squares regression. Both the sphere and the ellipsoid fit significantly better than the cone or the cylinder (p<0.001, F-test of correlation coefficients). There was no significant difference between the sphere and the ellipsoid fits (p>0.05, F-test of correlation coefficients).

We elected to model the mystacial pad as an ellipsoid rather than as a sphere because the ellipsoid has six free parameters (major radius, semi-major radius, minor radius and the radii thereof) that can be varied to generate different surface contours. This flexibility in shape will be important in future studies that aim to model natural whisking behavior, as the mystacial pad changes its curvature during the whisking cycle [9].

Mystacial pads were fit with seven parameters describing an ellipsoid: ellipsoid center (**c**), three radii *(r_a_, r_b_, r_c_)*, and three orientation angles defined by the major, semi-major and minor axis vectors *(θ_mp_, ϕ_mp_, ψ_mp_)*. Parameters were fit to each side of each rat's face using the whisker base-point locations *(x, y, z)* after normalizing for different head widths as described above. Ellipsoid parameters were calculated for each side of each rat using a least squares algorithm developed by Li and Griffiths [28] based on a Lagrange multiplier method.

The three radii obtained from the least squares fit yield an axis-aligned ellipsoid defined by the equation: 
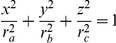
. This ellipsoid can then be rotated (equation [8]) and translated (by the ellipsoid center point, ***c***) into the 3D position and orientation required to align it with the mystacial pad surface. The resulting ellipsoidal fits for all rats are shown in Figure 2 of Results.

#### Whisker base-point position relative to the mystacial ellipsoid (r_BP_, θ_BP_, ϕ_BP_)

Having used the whisker base-points from the raw data to develop the underlying model for the mystacial pad, we next forced all whisker base-points *(x,y,z)* to lie on the ellipsoid surface. The mapping used spherical coordinates relative to the ellipsoid center (radius *r_BP_*, azimuth *θ_BP_*, inclination *ϕ_BP_*) and is shown in Figure 3A. Each base-point was adjusted in the radial direction so as to lie on the ellipsoid surface (*r_BP_*). The radius for a point on the surface of a given ellipsoid is given by equation [10] of Results.

#### Exclusion criteria for determining the fraction of the whisker that lies in a plane

A key assumption underlying the parameterization of whisker shape is that a significant fraction of the whisker's length lies in a single plane. A previous study, based on 105 whiskers, reported that the proximal-most 70% of a whisker is approximately planar [10]. Because the present study measured both 3D and 2D shape of 158 whiskers, the data set potentially provides the means to validate this result using a larger number of whiskers. One complication, however, is that the 3D scanner did not have the resolution to always image the most distal whisker regions. Therefore, the planar assumption was validated using only whiskers for which the 3D scanner captured a fraction of the whisker length greater than the fraction of the whisker computed to lie in the plane. Eighty-four whiskers met this criterion.

#### Whisker shape (s, a)

To find the best quadratic fit, we minimized the mean-square error between the curve 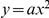 and the whisker 2D shape by permitting the whisker data to rotate and translate until it best matched the curve. Error was computed as the Euclidian distance between 50 equivalent arc lengths between the parabola and whisker. All whiskers were fit with an r^2^ greater than or equal to 0.99 for 93% of all whiskers scanned, and an r^2^ greater than or equal to 0.90 for 99% of all whiskers scanned (mean r^2^: 0.9945, standard deviation of r^2^: 0.022). To convert between the quadratic fit parameters (*s* and *a*) to Cartesian *x* coordinate requires finding a function *f* such that: 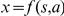. Finding this function is discussed in Supplementary Information Text S1.

#### Whisker orientation (θ, ϕ, ψ, and ζ)

Three whisker projection angles *(θ, ϕ, ψ)* were computed by projecting the linear portion of the 3D whisker onto each of the three Cartesian planes (*xy*, *xz*, and *yz*). Conventions for projection angle are shown in Figure 7 of Results. The linear portion of a 3D scanned whisker was defined as the largest arc length at which the maximum residual to a line (passing through the base-point and that arc length) was below 150 microns. Five whiskers (out of 158) were especially noisy, and this residual threshold had to be increased to 500 microns to produce a meaningful linear region. The linear region was then projected onto all three coordinate planes and angles relative to each coordinate axis calculated. All projection angles were defined so as to be independent of the side of the face.

Following standard convention, the projection angle *θ* defines the whisker protraction angle [4], [7], [10], [13], [14], [29], [30], [31], [32.]. Theta represents the angle obtained between the y-axis and a projection of the proximal (linear portion) of the whisker into the *xy*-plane. Theta is defined to increase as the rat protracts its whiskers, with 180° representing the whisker pointing rostrally and 0° representing the whisker pointing caudally (Figure 7B).

The projection angle *ψ* is computed as the angle between the *y*-axis and the projection of the linear whisker segment into the *yz*-plane (sagittal plane). As shown in Figure 7C, the angle *ψ* is 0° if the linear portion of the whisker points caudally, 90° if it points dorsally, 180° if it points rostrally, and 270° if it points ventrally.

The projection angle *ϕ* represents a whisker rotation in the dorsal-ventral direction (coronal plane), and is computed as the angle between the *x*-axis and the projection of the linear portion of the whisker into the *xz*-plane. The angle ϕ is defined to increase for dorsal rotations, with +90° representing the whisker pointing straight up and −90° representing the whisker pointing straight down (Figure 7D).

The angle *ζ* was defined based on the planar region of the whisker. The whisker plane was defined as the plane that passed through the whisker's base-point, the end of the whisker's linear region, and the largest arc length at which the maximum residual to the plane was below 150 microns. Three especially noisy whiskers required an increased residual threshold of 400 microns to produce a meaningful planar region. The angle *ζ* was defined so that its sign indicated the curvature orientation direction (Figure 7E and 7F). With all other projection angles set to 0°, *ζ* equal to −90° means that the whisker is parallel to the *xy*-plane and curves concave backwards (caudal). At *ζ* equal to 0°, the whisker lies parallel to the *xz*-plane and curves concave downwards (ventral). At *ζ* equal to +90°, the whisker lies parallel to the *xy*-plane and curves concave forwards (rostral). At *ζ* equal to ±180°, the whisker lies parallel to the *xz*-plane and curves concave upwards (dorsal).

These conventions were chosen so that all positive values of *ζ* place the whisker so that its concave side is oriented towards the front of the rat. All negative values of *ζ* place the whisker with its concave side oriented towards the back of the rat.

### Quantification of error in the 3-dimensional scans

Resolution limits of the 3D scanner meant that the scan often did not capture the whisker's most distal region. The fraction of each whisker that was captured in the 3D scan was calculated as the ratio of the 3D scan whisker length to the 2D scan whisker length. The 2D scan was sufficiently high resolution (between 3 and 10 pixels/micron) that it captured all of the whisker tips and can be considered “ground truth” for whisker length. In general, the 3D scan captured 50%±30% (STD) of the 2D whisker length. This is sufficient to estimate the whisker's base-point location and angles of emergence, which require only the whisker's most proximal portion. The parameter most greatly affected by the limited 3D scanner resolution is the *ζ* angle, which requires estimation of the whisker plane. For example, if only 10% of the whisker is scanned in 3D, the data will most likely be linear, and a plane would be poorly conditioned. The *ζ* angle was therefore only computed when a well-conditioned plane could be found (84 out of 158 whiskers scanned in 3D).

### Equations relating whisker parameters to whisker identity

To combine the mystacial pad parameters and the whisker specific parameters across all rats into a single model, we found the underlying relationship between each of the eight whisker specific parameters (*θ_BP_, ϕ_BP_,* s, a, *θ, ϕ, ψ, ζ*) and two independent variables: row and column identity.

A two-way ANOVA was performed for each parameter with the row and column as possible factors to find identity-based relationships. To perform the analysis, each row was assigned an integer value between 1 and 5 (increasing from A to E) and each column an integer value between 1 and 6 (increasing from caudal to rostral). Greek columns were associated with an adjacent row (α: A, β: B, γ: C, δ: D) and assigned a column integer value of 0. For parameters that had only the row or the column as a significant predictor (p<0.001), a least squares regression analysis was used to test four different underlying relationships between the parameter and the position variable of interest. The four types of models tested were: polynomial, rational, power, and exponential functions (see Supplementary Information Text S1 and Table S2 for a comprehensive list of equations). For parameters that had both the row and the column as significant factors (ANOVA, p<0.001), only multivariate regression was performed to generate the underlying relationship. These parameters were tested with polynomial relationships. To avoid overfitting, a higher-order or non-polynomial fit was chosen as the appropriate fit only if it was significantly better than the linear fit (F-test of the correlation coefficients, p<0.001). The final equation types used in our analysis included linear, exponential and 2D linear (equations shown in Table 4).

**Table 4:** Functions for the idealized whisker array.

	Fit Type	Equation
One input parameter	Linear	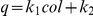 OR 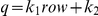
	Exponential	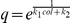
Two input parameters	2D Linear	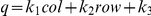

*q* indicates the parameter value being tested. *k* represents the coefficients found from regression.

The average mystical pad ellipsoid was calculated from the right and left ellipsoids of three rats. Parameters from left-side ellipsoids were mirror-imaged across the *yz*-plane so that they could be averaged with parameters from right-side ellipsoids. After averaging all seven parameters, the resulting average ellipsoid was mirrored back across the *yz*-plane to generate symmetric, average ellipsoids on both right and left sides. Finally, these averaged ellipsoids were rotated about the global *x*-axis to ensure that the mean of the final base-point row-planes was parallel to the *xy*-plane (by an angle of 8.72°), therefore following the convention set in Results for the head orientation. The final values of ellipsoid parameters after this rotation are shown in Table 2.

### Simulating whisker-object contact patterns

#### Establishing the whisker array, object parameters, and rotation planes for the whisker rows

Whisker-object contact patterns were simulated using three different sets of parameters for the whisker array: (1) the parameters specified in Table 3, corresponding to the actual morphology of the whisker array; (2) the parameters from Table 3 except that all *ζ* angles were set equal to −90 degrees (all whiskers are convex backward); or (3) the parameters from Table 3 except that all ζ angles were set equal to +90 degrees (all whiskers are concave forward). The array was then placed in the standard 3D position and orientation shown in Figure 1.

A virtual object (a cylinder) was positioned symmetrically 5 mm in front of the rat's nose (Figure 11A in Results). Cylinder radii were chosen so that the change in curvatures was approximately linear, resulting in radii of plus and minus 60, 80, 120, 180, 280, 700 and infinity (flat) mm.

Next, a rotation plane for each row of whiskers was defined. The rotation plane is the plane in which the whiskers of a given row will rotate. To determine the rotation plane, each whisker was discretized into 99 segments (100 points along its length). The rotation plane is then defined by three points: the base-point of the rostral-most whisker in the row, the base-point of the caudal-most whisker in the row, and the average of all 100 3D discretized coordinates of every whisker in the row. This generates a plane that passes through the whisker base-points and through the average 3D position of all the whiskers in a given row.

#### Simulation of whisker kinematics and calculation of contact angle

**Figure 14:**
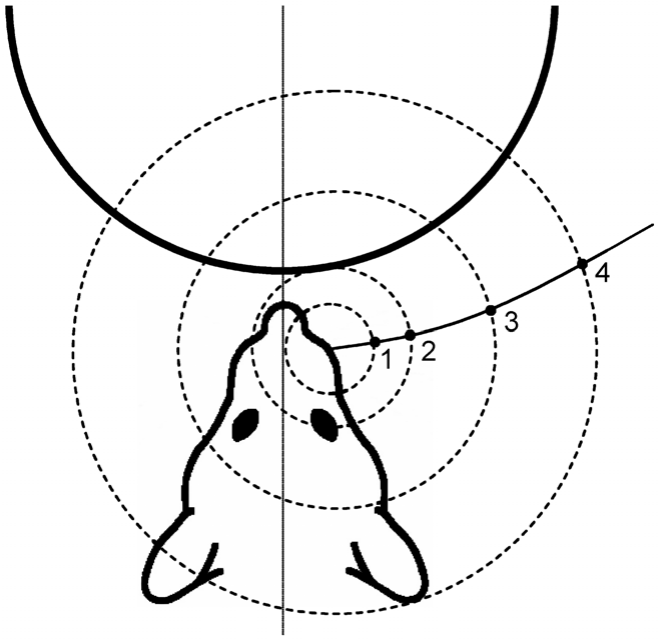
Simulation of whisker-object collisions. Circles are centered on the whisker base-point and constrain the movement of points on each whisker within a given rotation plane. Four locations along the whisker are indicated as black dots. The object (a cylinder) is indicated as a heavy black line. Only one whisker is shown and its length has been exaggerated for visual clarity.

In principle, the initial angles of contact that the whiskers made with the cylinder could have been determined by explicitly running simulations of the forward-kinematics. In this procedure, each whisker would be incrementally rotated forward within its plane of rotation at each time step, and the angles at which the whisker first collided with the object determined. In practice, however, this procedure is computationally expensive and time prohibitive. Instead, geometric relationships between the array and the object can be exploited to obtain the same results as would have been generated via a full kinematic simulation. Three steps are involved:

First, a set of circles is defined at each of the 100 discretized points along each whisker. Each circle is coplanar with the rotation plane, centered on the whisker's base-point, and has a radius equal to the distance between the base-point and each of the 100 points along the whisker. Figure 14 schematizes a set of circles for one whisker whose rotation plane is parallel to the *xy*-plane. For visual clarity, only four of 100 circles are shown, and the whisker's length has been greatly exaggerated. Each circle represents all possible locations of a particular point on the whisker rotating in the rotation plane.

Second, the intersection points of each circle and the object are calculated. Each circle and the object will intersect either at zero points (circle 1 in Figure 14), one point (circle 2 in Figure 14) or two points (circles 3 and 4 in Figure 14). In the case that the circle intersects the object at two points, the intersection point that requires the whisker to be at a positive angle less than 180 degrees is chosen as the point of initial contact.

Finally, the angle between the negative y-axis and the vector pointing from the whisker base-point to the end of the linear portion of the whisker is determined. The smallest of these angles (indicating the first point to contact) is taken as the initial contact angle.

## Supporting Information

Dataset S1 Matlab functions to generate the “RatMap”.
(0.01 MB ZIP)

Figure S1 Cesàro linear fit coefficients to the whisker 2D shape. (A) The general shape of any given whisker can be inferred from the coefficients of its fit to the equation κ (s) = *A*s+*B*. Abbreviations (in red) describe how the curvature changes from base to tip along the length of a whisker for special regions of the coefficient space, and are defined in the table of abbreviations (Table S1). A graphical example of each type of curvature is provided in the plot. Diagonal dotted lines indicate *A* = *−B* and *A* = *B*. (B) Coefficients *A* versus *B* for whiskers of normalized arc length. The best fit linear approximation is shown in dark gray along with the corresponding equation. The two coefficient coefficients were highly correlated (correlation coefficient r = −0.867), with a statistically significant relationship (p<0.001). Gray line depicts this linear fit. The dashed lines represent *A* = *−B* and *A* = *B*. Asterisk indicates one outlier data point that was outside the plot boundaries.
(0.51 MB TIF)

Figure S2 Dependence of *Φ_e_* and *φ_e_* on whisker identity (row or column location in the array). (A–B) The angles *Φ_e_* and *φ_e_* obtained from all three rats, plotted against either row or column as indicated. Different marker colors (black, gray, and light gray) indicate rat of origin and demonstrate that no significant differences were found across rats.
(0.19 MB TIF)

Table S1 Cesàro notation and interpretation of different coefficient combinations.
(0.03 MB DOC)

Table S2 All models tested for parameter-identity relationships.
(0.05 MB DOC)

Text S1 Supplementary information.
(0.06 MB DOC)

## References

[1] Vincent SB (1912). The function of vibrissae in the behavior of the white rat.. Behavior Monogr.

[2] Welker WI (1964). Analysis of sniffing of the albino rat.. Behaviour.

[3] Dörfl J (1982). The Musculature of the Mystacial Vibrissae of the White-Mouse.. J Anat.

[4] Harvey MA, Bermejo R, Zeigler HP (2001). Discriminative whisking in the head-fixed rat: optoelectronic monitoring during tactile detection and discrimination tasks.. Somatosens Mot Res.

[5] Krupa DJ, Matell MS, Brisben AJ, Oliveira LM, Nicolelis MAL (2001). Behavioral properties of the trigeminal somatosensory system in rats performing whisker-dependent tactile discriminations.. J Neurosci.

[6] Guic-Robles E, Valdivieso C, Guajardo G (1989). Rats can learn a roughness discrimination using only their vibrissal system.. Behav Brain Res.

[7] Carvell GE, Simons DJ (1990). Biometric Analyses of Vibrissal Tactile Discrimination in the Rat.. J Neurosci.

[8] Polley DB, Rickert JL, Frostig RD (2005). Whisker-based discrimination of object orientation determined with a rapid training paradigm.. Neurobiol Learn Mem.

[9] Bermejo R, Friedman W, Zeigler HP (2005). Topography of whisking II: Interaction of whisker and pad.. Somatosens Mot Res.

[10] Knutsen PM, Biess A, Ahissar E (2008). Vibrissal kinematics in 3D: Tight coupling of azimuth, elevation, and torsion across different whisking modes.. Neuron.

[11] Brecht M, Preilowski B, Merzenich MM (1997). Functional architecture of the mystacial vibrissae.. Behav Brain Res.

[12] Haidarliu S, Ahissar E (2001). Size gradients of barreloids in the rat thalamus.. J Comp Neurol.

[13] Towal RB, Hartmann MJZ (2008). Variability in velocity profiles during free-air whisking behavior of unrestrained rats.. J Neurophysiol.

[14] Towal RB, Hartmann MJ (2006). Right-left asymmetries in the whisking behavior of rats anticipate head movements.. J Neurosci.

[15] Meyer SL (1975). Data analysis for scientists and engineers.

[16] Dickinson MH, Farley CT, Full RJ, Koehl MAR, Kram R (2000). How animals move: An integrative view.. Science.

[17] Chiel HJ, Ting LH, Ekeberg O, Hartmann MJZ (2009). The Brain in Its Body: Motor Control and Sensing in a Biomechanical Context.. J Neurosci.

[18] Jacquin MF, Zeigler HP (1982). Trigeminal Orosensory Deafferentation Disrupts Feeding and Drinking Mechanisms in the Rat.. Brain Res.

[19] Ahl AS (1986). The role of vibrissae in behavior - A status review.. Vet Res Commun.

[20] Lawing AM, Polly PD (2010). Geometric morphometrics: recent applications to the study of evolution and development.. J Zool.

[21] Berdnikovs S, Bernstein M, Metzler A, German RZ (2007). Pelvic growth: Ontogeny of size and shape sexual dimorphism in rat pelves.. J Morphol.

[22] Caumul R, Polly PD (2005). Phylogenetic and environmental components of morphological variation: Skull, mandible, and molar shape in marmots (Marmota, Rodentia).. Evolution.

[23] Ghazanfar AA, Nicolelis MAL (1997). Nonlinear processing of tactile information in the thalamocortical loop.. J Neurophys.

[24] Drew PJ, Feldman DE (2007). Representation of moving wavefronts of whisker deflection in rat somatosensory cortex.. J Neurophysiol.

[25] Shimegi S, Akasaki T, Ichikawa T, Sato H (2000). Physiological and anatomical organization of multiwhisker response interactions in the barrel cortex of rats.. J Neurosci.

[26] Shimegi S, Ichikawa T, Akasaki T, Sato H (1999). Temporal characteristics of response integration evoked by multiple whisker stimulations in the barrel cortex of rats.. J Neurosci.

[27] Salinas E (2006). How behavioral constraints may determine optimal sensory representations.. PLoS Biol.

[28] Li QD, Griffiths JG (2004). Least squares ellipsoid specific fitting.. Geom Model Process.

[29] Bermejo R, Vyas A, Zeigler HP (2002). Topography of rodent whisking - I. Two-dimensional monitoring of whisker movements.. Somatosens Mot Res.

[30] Grant RA, Mitchinson B, Fox CW, Prescott TJ (2009). Active Touch Sensing in the Rat: Anticipatory and Regulatory Control of Whisker Movements During Surface Exploration.. J Neurophysiol.

[31] Mitchinson B, Martin CJ, Grant RA, Prescott TJ (2007). Feedback control in active sensing: rat exploratory whisking is modulated by environmental contact.. Proc R Soc Lond B Biol Sci.

[32] Sachdev RNS, Sato T, Ebner FF (2002). Divergent movement of adjacent whiskers.. J Neurophysiol.

