# Supplementary figures and images for: The Morphology of the Rat Vibrissal Array: A Model for Quantifying Spatiotemporal Patterns of Whisker-Object Contact
Source: PLoS Comput Biol. 2011 Apr 7;7(4):e1001120. doi: 10.1371/journal.pcbi.1001120 (PMC3072363; doi:10.1371/journal.pcbi.1001120)

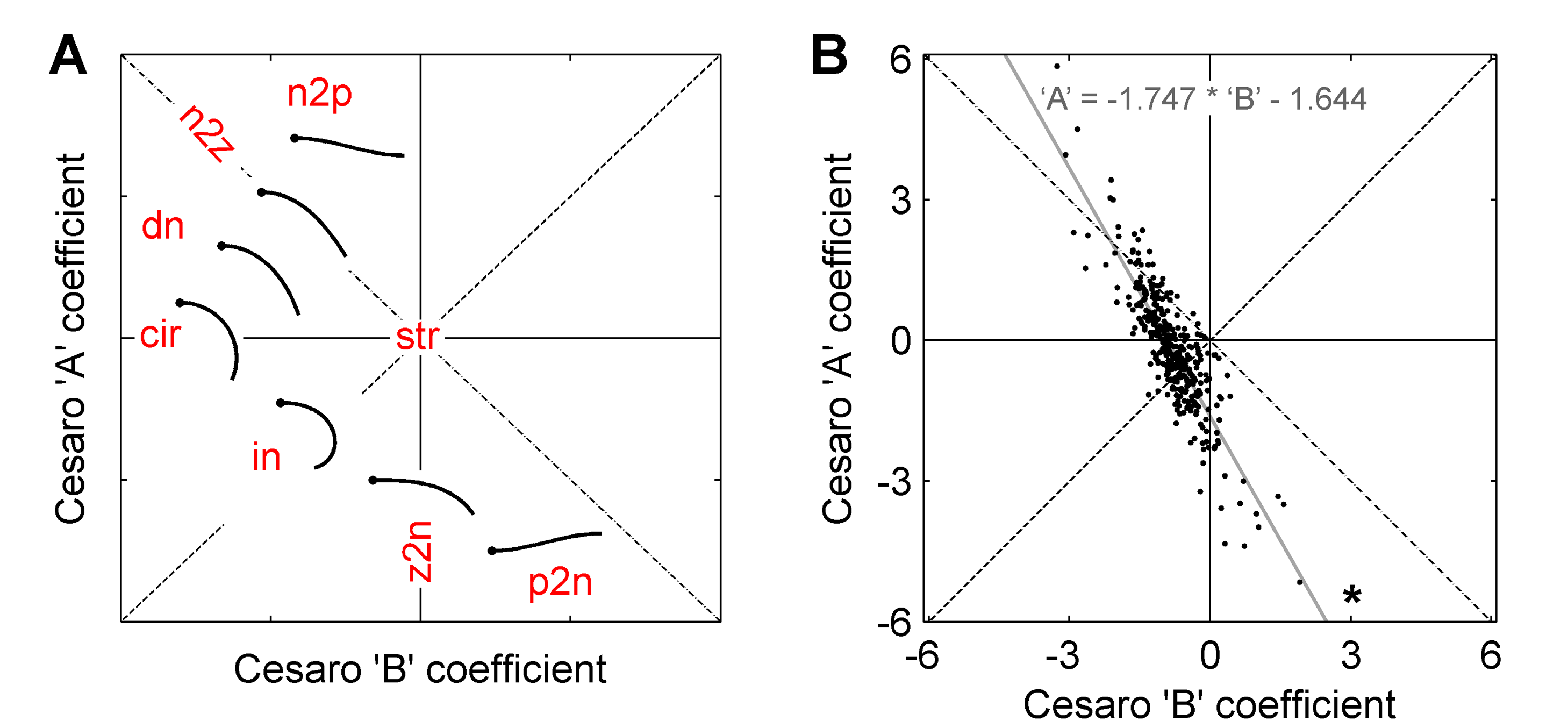

Supplement: Figure S1 — Cesàro linear fit coefficients to the whisker 2D shape. (A) The general shape of any given whisker can be inferred from the coefficients of its fit to the equation κ (s) = As+B. Abbreviations (in red) describe how the curvature changes from base to tip along the length of a whisker for special regions of the coefficient space, and are defined in the table of abbreviations (Table S1). A graphical example of each type of curvature is provided in the plot. Diagonal dotted lines indicate A = −B and A = B. (B) Coefficients A versus B for whiskers of normalized arc length. The best fit linear approximation is shown in dark gray along with the corresponding equation. The two coefficient coefficients were highly correlated (correlation coefficient r = −0.867), with a statistically significant relationship (p<0.001). Gray line depicts this linear fit. The dashed lines represent A = −B and A = B. Asterisk indicates one outlier data point that was outside the plot boundaries. (0.51 MB TIF) [file pcbi.1001120.s002.tif]

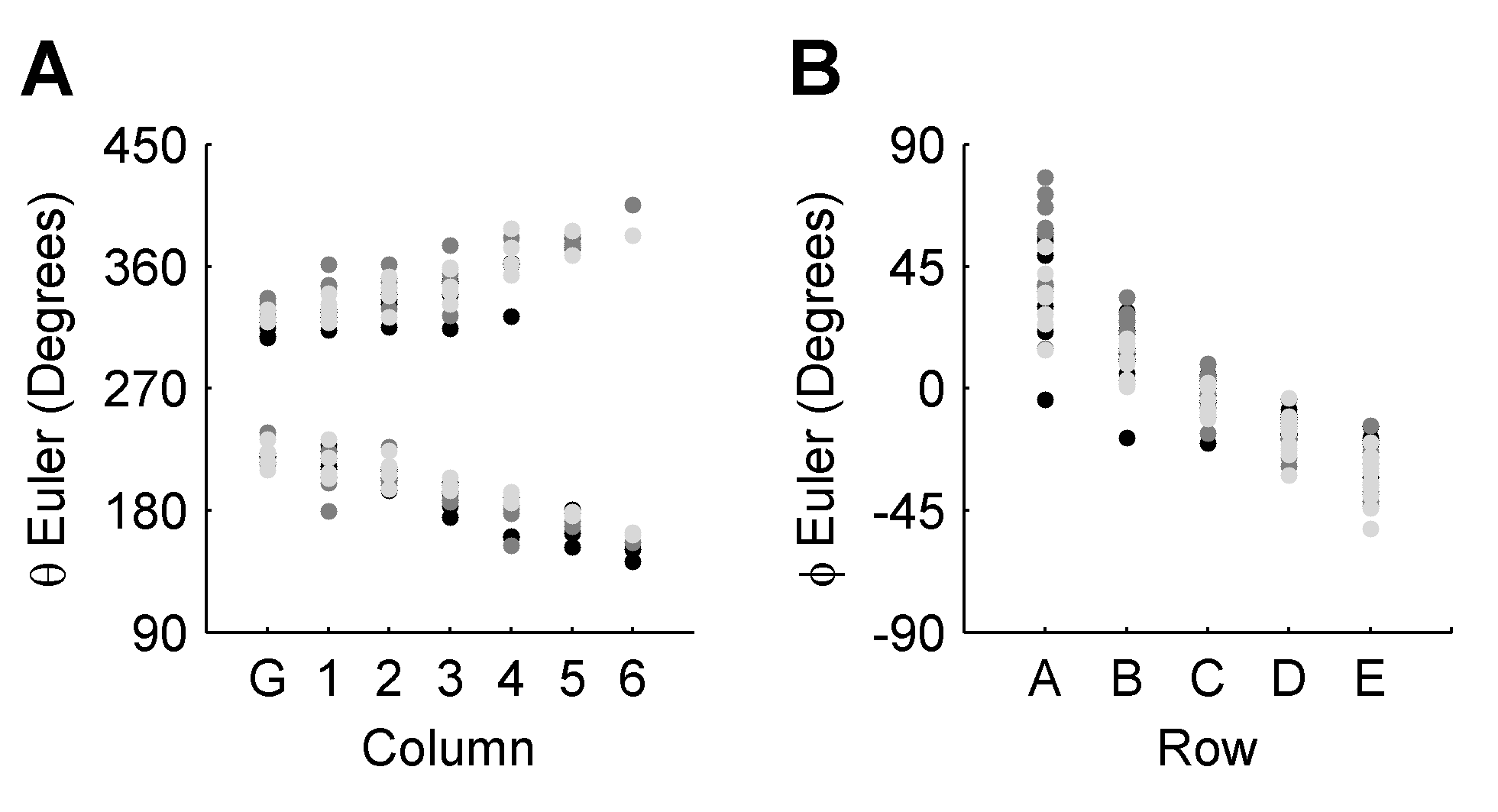

Supplement: Figure S2 — Dependence of Φe and φe on whisker identity (row or column location in the array). (A–B) The angles Φe and φe obtained from all three rats, plotted against either row or column as indicated. Different marker colors (black, gray, and light gray) indicate rat of origin and demonstrate that no significant differences were found across rats. (0.19 MB TIF) [file pcbi.1001120.s003.tif]
