# Supplementary material for: The Morphology of the Rat Vibrissal Array: A Model for Quantifying Spatiotemporal Patterns of Whisker-Object Contact
Source: PLoS Comput Biol. 2011 Apr 7;7(4):e1001120. doi: 10.1371/journal.pcbi.1001120 (PMC3072363; doi:10.1371/journal.pcbi.1001120)
Supplement: Table S1 — Cesàro notation and interpretation of different coefficient combinations. (0.03 MB DOC) [file pcbi.1001120.s004.doc]

**Table S1. Cesàro notation and interpretation of different coefficient combinations**

| **Notation:** | **Meaning:** | **‘A’  coefficient** | **‘B’  coefficient** | **Interpretation:** |
| --- | --- | --- | --- | --- |
| *str* | Straight | A = 0 | B = 0 | Curvature is zero along the entire whisker length, meaning the whisker is a straight line. |
| *cir* | Circular | A = 0 | B ≠ 0 | Curvature is constant so that the whisker shape is a segment of a circle |
| *z2n* | zero to negative | A < 0 | B = 0 | Curvature is zero (straight) at the base and linearly decreases towards the tip. |
| *n2z* | negative to zero | A = - B | B < 0 | Curvature starts out negative at the base and linearly increases along the whisker to become zero (straight) at the tip. |
| *in* | Increasingly negative | A < 0 | B < 0 | Curvature starts out negative at the base and linearly becomes increasingly negative towards the tip. |
| *dn* | decreasingly negative | A > 0  A < -B | B < 0 | Curvature starts out negative at the base and linearly becomes less negative towards the tip, but does not reach zero. |
| *n2p* | negative to positive | A > -B | B < 0 | Curvature starts out negative at the base and linearly increases along the whisker to become positive before the tip. The shape is similar to part of a sigmoid curve. |
| *p2n* | positive to negative | A < -B | B > 0 | Curvature starts out positive at the base and linearly decreases along the whisker to become negative before the tip. The shape is similar to part of a sigmoid curve. |
