# Supplementary material for: The Morphology of the Rat Vibrissal Array: A Model for Quantifying Spatiotemporal Patterns of Whisker-Object Contact
Source: PLoS Comput Biol. 2011 Apr 7;7(4):e1001120. doi: 10.1371/journal.pcbi.1001120 (PMC3072363; doi:10.1371/journal.pcbi.1001120)
Supplement: Table S2 — All models tested for parameter-identity relationships. (0.05 MB DOC) [file pcbi.1001120.s005.doc]

**Table S2. All models tested for parameter-identity relationships**

|  | ***Fit Type:*** | ***Equation:*** |
| --- | --- | --- |
| Single identity parameter | Linear |  |
|  | Quadratic | $q=k_{1}col^{2}+k_{2}col+k_{3}$ |
|  | Cubic |  |
|  | Rational (A) | $q=k_{1}/(col+k_{2})$ |
|  | Rational (B) | $q=(k_{1}col+k_{2})/(col+k_{3})$ |
|  | Power Law (A) | $q=k_{1}col^{k_{2}}$ |
|  | Power Law (B) | $q=k_{1}col^{k_{2}}+k_{3}$ |
|  | Exponential (A) | $q=k_{1}e^{k_{2}col}$ |
|  | Exponential (B) | $q=k_{1}e^{k_{2}col}+k_{3}e^{k_{4}col}$ |
|  | Exponential (C) | $q=e^{\frac{1}{k_{1}col+k_{2}}}$ |
| Two identity parameters | 2D Linear | $q=k_{1}col+k_{2}row+k_{3}$ |
|  | 2D Quadratic (A) | $q=k_{1}col^{2}+k_{2}col+k_{3}row+k_{4}$ |
|  | 2D Quadratic (B) | $q=k_{1}row^{2}+k_{2}col+k_{3}row+k_{4}$ |
|  | 2D Quadratic (C) | $q=k_{1}col^{2}+k_{2}col+k_{3}row^{2}+k_{4}row+k_{5}$ |

Note that in each equation for a single identity parameter, *col* could be replaced with *row*. *col:* column.
