# Supplementary material for: The Morphology of the Rat Vibrissal Array: A Model for Quantifying Spatiotemporal Patterns of Whisker-Object Contact
Source: PLoS Comput Biol. 2011 Apr 7;7(4):e1001120. doi: 10.1371/journal.pcbi.1001120 (PMC3072363; doi:10.1371/journal.pcbi.1001120)
Supplement: Text S1 — Supplementary information. (0.06 MB DOC) [file pcbi.1001120.s006.doc]

**SUPPLEMENTARY INFORMATION TEXT S1**

**S1.1 *Cesàro* fits to the 2D whisker shape**

Although the parabolic approximation is simple and intuitive, it is not a coordinate free description of whisker shape. The parabolic fit coefficients depend on the whisker orientation in the *xy* coordinate system. To describe whisker shape without reference to Cartesian coordinates, the 2D whisker shape was fit using a coordinate free or “intrinsic” representation of whisker curvature. In a coordinate free representation of a plane curve, one end of the curve is specified as the origin and the position of any point on the curve is specified by the arc length *s* traveled along the curve from the origin to reach that point . The curve shape is then described by specifying the curvature *κ(s)* at each point, as a function of the arc length *s*. Thus, the set of values $\left\{ s,\kappa\left(s\right) \right\}$ completely describes the curve intrinsically with respect to *s*, without reference to an external coordinate system.

In the absence of any *a priori* model for *κ(s)*, excellent fits to the data were obtained using a linear parameterization: $\kappa\left(s\right)=As+B$, where *s* is normalized by whisker length (*s* ranges from 0 to 1), and *(A, B)* are the linear best fit parameters. The fitting process involved discretizing each whisker arc length into 50 nodes. Values for *A* and *B* were found by minimizing the mean sum of squared Euclidean distances between the nodes of the normalized actual whiskers and the nodes of the coordinate free curves. Excellent fits were obtained using this approach, except in the rare case in which the whisker exhibited a strong curvature change near its middle, similar to an ‘s’ shape.

The sign of the curvature was defined using the direction of rotation of the tangent at two sequential points along the whisker curve. This convention always moved from a point closer to the whisker base to a point closer to the tip. Positive curvature was defined as a counter-clockwise rotation of the tangent. Negative curvature was defined as a clockwise rotation of the tangent.

With such a simple governing equation, it is possible to get a sense of the whisker shape simply by looking at the fit coefficients. Figure S1 shows a graphical representation of the fit coefficient parameter space along with example whisker curves. The Cesàro *A* coefficient determines the change in curvature along the arc length. The Cesàro ‘*B*’ coefficient determines the constant, underlying curvature. For example, a segment of a circle results in curve fits parameters where *A* is zero and *B* is non-zero. The examples in Figure S1A also demonstrate that the equation $\kappa\left(s\right)=As+B$ is able to fit a wide diversity of whisker shapes, even though there are only two free parameters. Labels are defined in Table S1 for the possible types of relations between Cesàro *A* and *B* coefficients for natural whisker shapes.

The resulting coordinate free parameter fits of whisker shape are shown in Figure S1B There was a strong correlation between the two fit coefficients (*r* = -0.867). The linear relationship between the two fit coefficients was statistically significant (p < 0.001). The majority of whiskers (51%) exhibited curvatures that were classified as increasingly negative (‘*in’*), and were closely clustered near the origin. The next largest grouping whisker fit coefficients were classified as decreasingly negative (‘*dn’*, 33%). Finally, the shapes classified as negative-to-positive (*‘n2p’*, 9%) and positive-to-negative (*‘p2n’*, 7%) made up the remaining observed whisker fit coefficient types.

1. do Carmo MP (1976) Differential geometry of curves and surfaces. Englewood Cliffs, N.J.: Prentice-Hall.

**S1.2 Converting between projection angles and Euler angles**

*S1.2.1 Equations that relate projection angles to Euler angles*

Two common conventions for describing the orientation of an object in a 3D coordinate system are projection angles and Euler angles. Euler angles are defined in terms of rotations, and are therefore likely to be useful for modeling whisker movements, but their physical meaning is relatively unintuitive. In contrast, projection angles have a very physically intuitive meaning – indeed, they are the angles typically measured in studies of rat whisking behavior – but do not lend themselves well to performing rotations in modeling work. Although the projection angles presented in the paper are intuitive and behaviorally relevant, they are cumbersome when rotating the whiskers from a standard orientation to the correct orientation for the model. Euler angles provide a more natural representation of the emergence angles when rotations are necessary.

In the case of the present study, the angles of emergence of the whiskers (i.e. the angles of the linear portion of the whisker) can be described either with three projection angles, or with two Euler angles and an order of rotation. Conversions between the projection angles and the Euler angles are provided in equations S1-3.

The transformation from the θ projection angle to the *θ_e_* Euler angle is simply an offset (Equation S1). The *φ_e_* Euler angle depends on both the φ and ψ projection angles as well as on the *θ_e_* Euler angle (Equation S2). For example, if the *θ_e_* Euler angle is 0°, then the whisker exists only in the *xz*-plane, and therefore the *ψ* projection angle does not exist. In contrast, when the *θ_e_* Euler angle is 90°, then the whisker exists only in the *yz*-plane, and the *φ* projection angle does not exist. These examples illustrate that the φ and ψ projection angles are not independent and provide an intuition for why only two Euler angles and an order of rotation are needed to describe the whiskers' angles of emergence.

The final Euler angle, *ζ_e_*, is used to describe the whisker’s orientation about its own axis. It is related to the projection angle *ζ* presented in the main text through an offset of 90° (see Equation S3).

$$\theta_{e}=\left\{\begin{matrix} \theta_{projection}-90^{\circ} & , & 90^{\circ}\leq\theta_{projection}\leq180^{\circ} & and & side=right \\ \theta_{projection}+270^{\circ} & , & 0^{\circ}\leq\theta_{projection}<90^{\circ} & and & side=right \\ (180^{\circ}-\theta_{projection})+90^{\circ} & , & 90^{\circ}\leq\theta_{projection}\leq180^{\circ} & and & side=left \\ 270^{\circ}-\theta_{projection} & , & 0^{\circ}\leq\theta_{projection}<90^{\circ} & and & side=left \end{matrix}\right.$$

(Equation S1)

$$\phi_{e}=-1*\left\{\begin{matrix} I. \arctan\left(\tan\left(\phi_{projection}\right)*\cos\left(\theta_{e}\right)\right) , 0^{\circ}\leq\theta_{e}\leq45^{\circ} \\ II. \arctan\left(\tan\left(180^{\circ}-\psi_{projection}\right)*\sin\left(\theta_{e}\right)\right) , 45^{\circ}<\theta_{e}\leq90^{\circ} \\ III. \arctan\left(\tan\left(180^{\circ}-\psi_{projection}\right)*\sin\left(180^{\circ}-\theta_{e}\right)\right) , 90^{\circ}<\theta_{e}\leq135^{\circ} \\ IV. \arctan\left(\tan\left(\phi_{projection}\right)*\cos\left(180^{\circ}-\theta_{e}\right)\right) , 135^{\circ}<\theta_{e}\leq225^{\circ} \\ V. \arctan\left(\tan\left(\psi_{projection}\right)*\sin\left(\left|180^{\circ}-\theta_{e}\right|\right)\right) , 225^{\circ}<\theta_{e}\leq270^{\circ} , 0^{\circ}\leq\psi_{projection}\leq90^{\circ} \\ VI. \arctan\left(\tan\left(\psi_{projection}-360^{\circ}\right)*\sin\left(\left|180^{\circ}-\theta_{e}\right|\right)\right) , 225^{\circ}<\theta_{e}\leq270^{\circ} , 270^{\circ}\leq\psi_{projection} \\ VII. \arctan\left(\tan\left(\psi_{projection}\right)*\sin\left(360^{\circ}-\theta_{e}\right)\right) , 270^{\circ}<\theta_{e}\leq315 , 0^{\circ}\leq\psi_{projection}\leq90^{\circ} \\ VIII. \arctan\left(\tan\left(\psi_{projection}-360^{\circ}\right)*\sin\left(360^{\circ}-\theta_{e}\right)\right) , 270^{\circ}<\theta_{e}\leq315^{\circ} , 270^{\circ}\leq\psi_{projection} \\ IX. \arctan\left(\tan\left(\phi_{projection}\right)*\cos\left(360^{\circ}-\theta_{e}\right)\right) , 315^{\circ}<\theta_{e}<360^{\circ} \end{matrix}\right.$$

(Equation S2)

$$\zeta_{e}=\left\{\begin{matrix} \zeta+90^{\circ} , side=right \\ 90^{\circ}-\zeta, side=left \end{matrix}\right.$$

(Equation S3)

*S1.2.2 Euler angles vary smoothly with whisker row-column identity*

Average Euler angles describing the whiskers' angles of emergence are shown in Figures S2A-B. Similar to the projection angles (Figure 8 in *Results*), the Euler angles vary smoothly across the array. The angle *θ_e_* varies most strongly with column, with little variation as a function of row. In contrast, *φ_e_* varies most strongly with row and varies little with column. The Euler angle *ζ_e_* is merely a shifted version of the projection angle *ζ* (Figure 8D and H) and thus is not shown.

**S1.3 Functional relation between the Cartesian coordinate x and parameters of the parabolic fit**

Two-dimensional whisker shape is parameterized using whisker length, *s*, and the coefficient of the quadratic term, *a*. It was necessary to find a relationship between the Cartesian coordinates of a point on the 2D whisker *(x, y)* and the arc length and parabolic coefficient of that same point. Because the relationship between x and y is the parabolic equation *y = ax^2^*, it remains only to find a function, *f*, such that $x=f(a,s)$.

One approach is to take the equation for arc length $s=\int_{a}^{b} \sqrt{1+\left(\frac{dy}{dx}\right)^{2}}dx$, integrate it and then solve the resulting equation for $\frac{dy}{dx}$. However, the right hand side of that equation integrates to a function from which it is not trivial to isolate $\frac{dy}{dx}$.

Instead, arc lengths were calculated over a set of *x* values and *a* values to create a surface. The surface was fit with a two-dimensional, fifth degree polynomial such that $x=f(a,s)$, shown in equation S4.

$$\begin{matrix} x=f(a,s)=-1.4+133.7a+1.27s-4334a^{2}-7.3as-0.01s^{2}+62930a^{3}+72.2a^{2}s-0.2as^{2} \\ -407600a^{4}-565.2a^{3}s+1.6a^{2}s^{2}+966000a^{5}+1706a^{4}s-4.8a^{3}s^{2} \end{matrix}$$

(Equation S4)

This polynomial varied smoothly, and had a correlation coefficient of *r = 0.9993.* Thus there was negligible error in the calculation of (*x, y*) from a given *a* and *s*.

**S1.4 All equations tested to relate whisker parameters to whisker identity**

As described in *Materials and Methods,* fits to each parameter were tested with four types of models between the parameter and row, column or both row and column. Polynomial, rational, power law and exponential models were tested for single identity parameter (row or column) relationships. Polynomial relationships were tested for two identity parameter relationships (row and column). The different relationships tested are shown in Table S2.
